# Swim, baby, swim: the active dispersal scenario of juvenile North Pacific loggerhead turtles revealed by historical satellite tracking data and novel operational oceanography products

**DOI:** 10.1186/s40462-025-00562-5

**Published:** 2025-06-10

**Authors:** Philippe Gaspar, Julien Temple-Boyer, Dana K. Briscoe, Masanori Kurita, Denise M. Parker, Jeffrey J. Polovina, Marc R. Rice, Tomomi Saito, George H. Balazs

**Affiliations:** 1 Mercator Ocean International, Toulouse, France; 2 Doerr School of Sustainability, Woods Institute for the Environment, Stanford University, Stanford, CA USA; 3 Institute of Marine Sciences, University of California Santa Cruz, Santa Cruz, CA USA; 4 Port of Nagoya Public Aquarium, Nagoya Port Foundation, Nagoya, Japan; 5 Golden Honu Services of Oceania, Newport, OR USA; 6 Pacific Islands Fisheries Science Center, NOAA (Retired), Honolulu, HI USA; 7 HPA Sea Turtle Research Program, Kamuela, HI USA; 8 Usa Marine Biological Institute, Kochi University, Tosa, Kochi Japan; 9 Golden Honu Services of Oceania, Honolulu, HI USA

**Keywords:** Loggerhead sea turtle, North Pacific Ocean, Juvenile pelagic phase, Active dispersal, Orientation, Migration

## Abstract

**Background:**

How juvenile sea turtles disperse during their first years at sea, known as the “lost years”, remains enigmatic. The oceanic circulation is known to play a major role, but the impact of the swimming activity is poorly understood, largely because juvenile tracking experiments rarely cover a significant fraction of the lost years’ period. In addition, errors in commonly used ocean current estimates make it difficult to properly separate, in tracking data, the effect of the swimming activity from that of the drift velocity. In this paper, we re-analyze the largest extant tracking data set concerning juvenile North Pacific (NP) loggerhead turtles (*Caretta caretta*), attempting to more precisely characterize their lost years’ swimming activity.

**Methods:**

Juvenile loggerhead trajectories are jointly analyzed with surface drifter trajectories from the Global Drifter Program and novel operational oceanography products from the Copernicus Marine service. Combining these data sets, we present a new method to reliably separate, at least on the large scale, the turtles swimming velocity from the drift velocity which includes the impact of the current, the wind and the waves.

**Results:**

Results reveal that the smallest juveniles perform large seasonal north-south migrations while drifting eastwards with ocean currents. As they grow larger, many individuals are observed to change behavior. While keeping their meridional seasonal migrations, they initiate their homing journey swimming vigorously westwards towards their natal area (Japan), against prevailing currents. The juvenile NP loggerheads’ swimming activity is thus best described as a series of Drifting then Homing Seasonal Migrations. High interindividual synchronicity is observed during these migrations, especially around the fall equinox when individuals start swimming southwards.

**Conclusion:**

While open-ocean dispersal of juvenile sea turtles is known to be largely governed by ocean currents, our results demonstrate that juvenile loggerheads’ dispersal in the NP is also largely shaped by their well-organized large-scale swimming activity which involves ample seasonal migrations and vigorous homeward movements against adverse currents. Such an active swimming strategy comes with high energy expenditure probably balanced by increased foraging success. Analysis of forthcoming juvenile tracking experiments with our new data processing method should help reveal if juveniles from other sea turtle populations or species have evolved similar swimming strategies.

**Supplementary Information:**

The online version contains supplementary material available at 10.1186/s40462-025-00562-5.

## Background

Conservation of endangered species requires knowledge of where individuals disperse during their different life stages. Migration corridors and key habitats must indeed be identified to prioritize the implementation of protection measures. This is no easy task for sea turtles which are wide-ranging migrants displaying multiple ontogenetic habitat changes. In particular, from the moment they leave their natal beach, hatchlings remain largely cryptic until they reappear either as large juveniles recruiting to neritic areas, or as young adults coming back to their natal area to reproduce for the first time [1]. During that long period of several years to several decades (often called the “lost years”), juveniles disperse in the open ocean. They do so under the combined effects of their, largely unknown, swimming activity and the ocean currents. Carr [2], first hypothesized that the role of the swimming activity was negligible compared to that of currents. This “passive drift” hypothesis was based on the observation that juvenile loggerhead turtles (*Caretta caretta*) from Florida rookeries were seen at progressively larger sizes in areas following the clockwise water circulation around the North Atlantic subtropical gyre, going from Florida to the Azores, then Madeira and the Canary Islands to finally come back to the Caribbean area with the North Equatorial current.

Over the years, this passive drift model received support from numerous studies showing that the spatial distribution of juvenile sea turtles, of different species and in different ocean basins, was broadly consistent with down current transport from their nesting beaches [3–7]. But evidence also accumulated that juvenile sea turtles are far from being totally passive [8–11]. It became clear that, even if passive drift models are able to provide crude first-order approximations of juveniles’ spatial dispersal patterns [12–16], they fail to explain more detailed dispersal features. For example, the passive drift model displays the following shortcomings when applied to juvenile Florida loggerheads:

1. Passive drift does not explain dispersal timing between Florida and the Azores. Loggerheads born in Florida reach the Azores archipelago typically within 3 years [17], Passive drifters do so much more rapidly: their modal drift time from Florida to the Azores, is only 15 months [18].
2. Unlike envisioned by Carr, ocean circulation does not usually drive passive drifters all the way around subtropical gyres. It rather makes many of them converge inside these gyres, just like plastic debris do [12, 19, 20]. A passively drifting Florida loggerhead is thus more likely to end up circling well inside the North Atlantic subtropical gyre than to cross the whole North Atlantic basin and then drift back towards Florida.
3. Passive drift can lead passive juveniles into lethally cold waters, especially when they circulate along the northern edge of the subtropical gyre during wintertime [21, 22]. Strictly passive juveniles would thus have low chances of survival.
4. Passive drift does not explain why some oceanic juvenile loggerheads are observed to display meridional seasonal migrations [23, 24] or to return towards their natal area at latitudes where they do not benefit from favorable equatorial currents but rather encounter weak or opposing currents [23, 25]

The absence of any swimming activity in the passive drift model is clearly the cause of these shortcomings. Different types of swimming behaviors triggered by different sensory stimuli have been identified in sea turtles [20, 26] but, as of today, there is not a single sea turtle population for which a complete juvenile swimming scenario is known, and fully corrects for the shortcomings of the passive drift model. A partial solution, however, has been proposed for Florida loggerheads. Series of laboratory experiments indeed showed that hatchlings from that nesting population have an innate “magnetic map” in which different regional magnetic field values elicit changes in swimming direction at different locations around the North Atlantic subtropical gyre [21, 27–29]. Numerical experiments [22, 30] then suggested that simulated turtles driven by realistic high-resolution surface currents and weak (a few kilometers per day) swimming movements magnetically oriented as observed, were more likely than purely passive turtles to remain in warm waters and progress along their migration pathway around the subtropical gyre. This would largely correct for the above-mentioned shortcomings (2) and (3) of the passive drift model. This magnetically-guided swimming behavior however does not generate seasonal migrations, induce homing movements against dominant currents or explain why juveniles reach the Azores more slowly than passive drifters.

Other numerical dispersal simulations, including a swimming activity on top of passive drift, were also carried out for the West Pacific and North West Atlantic leatherback (*Dermochelys coriacea*) populations [31, 32]. The swimming activity was made of habitat-driven movements motivated by the need to find food and suitable water temperatures. Simulations showed that habitat-driven movements generate meridional seasonal migrations, reduce cold-induced mortality and yield transoceanic crossing times slower than those of passive drifters, in agreement with juvenile’s size observations on the eastern side of the two oceanic basins. However, these simulations did not deal with return movements towards nesting areas.

Altogether, these results suggest that a combination of different swimming behaviors triggered by different stimuli (magnetic, habitat-driven, …) is likely needed to correct for the inconsistencies of the passive drift model. The “winning combination” might not be simple, nor unique as different populations face different environmental challenges and must adapt accordingly. Although laboratory experiments and numerical simulations are certainly useful to help formulate and test possible swimming scenarios, direct observations are critically needed, if only to confirm these scenarios.

Unfortunately, direct observations of juvenile movements at sea are technically challenging and thus rare [33, 34]. They are mostly obtained from satellite-tracking experiments but, while large juveniles can be tracked over a year or so, tracking durations achieved with smaller individuals are systematically shorter (a few weeks to a few months) [9, 35, 36], mostly due to tags’ miniaturization and detachment issues [34, 37]. Juvenile tracking experiments are thus generally too short to allow for the reconstruction of the swimming activity over, at least, a significant fraction of the lost years’ period. In addition, swimming velocities derived from tracking data are subject to significant, but poorly assessed, errors. Tracking indeed does not provide a direct measure of the swimming velocity but a measure of the velocity over ground. This velocity is the sum of two, separately unknown, velocities: the swimming velocity and the drift velocity caused by the action of the currents, the wind and the waves on the turtle. Estimation of the swimming velocity thus requires subtracting an estimate of the drift velocity from the observed velocity over ground. As a consequence, swimming velocity estimation is impacted by the, often large and poorly quantified, errors affecting drift velocity estimates which are generally derived from operational ocean models. The usual assumption that the wind- and wave-induced components of the drift velocity are negligible compared to its current-induced component tend to make these errors larger. Such errors have a limited impact on adult sea turtle studies (their swimming velocities are generally large enough to be distinguished from model errors) but are problematic when analyzing the weaker swimming velocities of hatchlings and juveniles [38]. Therefore, to avoid mistaking model errors for a dummy swimming activity, trajectories of juvenile sea turtles are often jointly analyzed with trajectories of nearby passive surface drifters (oceanographic buoys) [10, 24, 39]. However, only short trajectory segments (typically a few days long) can be studied in this way as initially close drifter and turtle trajectories inevitably diverge and become uncorrelated. This approach is thus unsuitable for investigating the juveniles’ swimming activity over the long term.

In an attempt to correct for these problems, we develop here a new swimming velocity estimation method in which the swimming velocity estimation error is both reduced (by including the wind and wave effects) and better assessed (by making massive use of passive drifter data). This new estimation method is then used to revisit data from the exceptional juvenile loggerhead electronic tracking experiment initiated in 1997 by the NOAA Pacific Islands Fisheries Science Center (PIFSC) in collaboration with the Port of Nagoya Public Aquarium. During over 15 years, more than 200 juvenile loggerheads, with straight carapace lengths (SCL) ranging from 25 to 80 cm were tracked through the North Pacific (NP). Individual tracking durations are unusually long, often exceeding one year. The wide spatial and temporal coverage of this data set as well as the wide range of tracked individuals’ sizes makes it truly outstanding, likely appropriate to uncover a large part of the juvenile NP loggerheads’ swimming scenario. Data from this experiment were used in over 20 papers, addressing a range of questions including pelagic habitat and movement studies [8, 11, 40–44], movements towards Baja California neritic foraging grounds [45], diving behavior [46] and approaches to reducing bycatch [47, 48]. However, none of these papers presented a detailed investigation of the tracked turtles’ swimming velocity. This is achieved here. Our analysis reveals a previously undiscovered swimming scenario including two main swimming behaviors: an omnipresent seasonal north–south swimming activity and westward directed movements appearing, as a function of age, at latitudes where the ocean surface circulation is mostly eastwards. This second behavior likely takes juveniles back towards their natal area.

## Methods

### Swimming velocity estimation

#### Estimation principle

The velocity over ground, ${{\varvec{V}}}_{{\varvec{g}}}=({u}_{g},{v}_{g}$), of any buoyant object, permanently or occasionally present at the sea surface, is the sum of the object’s own velocity (${{\varvec{V}}}_{{\varvec{s}}}$) plus its drift velocity (${{\varvec{V}}}_{{\varvec{d}}}$) which results from the combined forces of the current, the wind and the waves acting upon the object:

1 $${{\varvec{V}}}_{{\varvec{g}}}= {{\varvec{V}}}_{{\varvec{s}}}+\boldsymbol{ }{{\varvec{V}}}_{{\varvec{d}}}$$

In the case of a turtle, ${{\varvec{V}}}_{{\varvec{s}}}$ is the swimming velocity. For a motor boat, it is the velocity induced by the action of the propeller and for passive drifters, like oceanographic buoys, sargassum or plastic debris, ${{\varvec{V}}}_{{\varvec{s}}}=0.$ Assuming that this object is tracked (usually satellite-tracked), and that its trajectory is known under the form of a discrete time series of surface positions ${\varvec{X}}\left({t}_{i}\right)=[x\left({t}_{i}\right),y\left({t}_{i}\right)]$ regularly sampled at time interval $\Delta t$, simple centered finite differences can be used to obtain discrete estimates of the velocity over ground (${\widehat{{\varvec{V}}}}_{{\varvec{g}}}$):

2 $${\widehat{{\varvec{V}}}}_{{\varvec{g}}}\left({t}_{i}+\frac{\Delta t}{2}\right)=\frac{\left[{\varvec{X}}\left({t}_{i+1}\right)-{\varvec{X}}\left({t}_{i}\right)\right]}{\Delta t}$$

If an estimate of the drift velocity (${\widehat{{\varvec{V}}}}_{{\varvec{d}}}$) can be obtained with the same time sampling, then Eq. (1) shows that the difference between ${\widehat{{\varvec{V}}}}_{{\varvec{g}}}$ and ${\widehat{{\varvec{V}}}}_{{\varvec{d}}}$ provides an estimate of the objects’ own velocity. This difference will be called the drift-corrected (DC) velocity:

3 $${{\varvec{V}}}_{{\varvec{D}}{\varvec{C}}}={\widehat{{\varvec{V}}}}_{{\varvec{s}}}={\widehat{{\varvec{V}}}}_{{\varvec{g}}}-{\widehat{{\varvec{V}}}}_{{\varvec{d}}}$$

When the impact of the wind and waves on the object’s movement is neglected, the drift velocity reduces to the Eulerian ocean current velocity, i.e. ${{\varvec{V}}}_{{\varvec{d}}}\boldsymbol{ }={{\varvec{V}}}_{{\varvec{c}}}$ (for more details see Sect. “Drift velocity estimation”). Therefore ${{\varvec{V}}}_{{\varvec{D}}{\varvec{C}}}$ can be viewed as an improved version of the current-corrected velocity previously defined by Gaspar et al. [49], or the heading velocity of Girard et al. [50].

#### Estimation error

${{\varvec{V}}}_{{\varvec{D}}{\varvec{C}}}$ Is obviously not a perfect estimate of the object’s own velocity. Using the generic notation $=\widehat{s}+{s}{\prime}$, where $\widehat{s}$ is the measured or estimated value of any variable $s$ and ${s}{\prime}$ is its error, (1) can be rewritten:

4 $${{\varvec{V}}}_{{\varvec{D}}{\varvec{C}}}={\widehat{{\varvec{V}}}}_{{\varvec{s}}}={\widehat{{\varvec{V}}}}_{{\varvec{g}}}-{\widehat{{\varvec{V}}}}_{{\varvec{d}}}=\boldsymbol{ }\boldsymbol{ }{{\varvec{V}}}_{{\varvec{s}}}+\boldsymbol{ }{({{\varvec{V}}}_{{\varvec{d}}}^{\boldsymbol{^{\prime}}}}_{\boldsymbol{ }}-{{\varvec{V}}}_{{\varvec{g}}}^{\boldsymbol{^{\prime}}})$$

This shows that ${{\varvec{V}}}_{{\varvec{D}}{\varvec{C}}}$ is the true object’s velocity (the swimming velocity in the sea turtle case) plus an error term, hereafter called the estimation noise, which includes the estimation errors affecting the drift velocity ${({{\varvec{V}}}_{{\varvec{d}}}^{\boldsymbol{^{\prime}}}}_{\boldsymbol{ }})$ and the velocity over ground $({{\varvec{V}}}_{{\varvec{g}}}^{\boldsymbol{^{\prime}}})$.

${{\varvec{V}}}_{{\varvec{g}}}^{\boldsymbol{^{\prime}}}$ is easily quantified in the usual case where positions ${\varvec{X}}\left({t}_{i}\right)$ are obtained from the Argos satellite tracking system. Indeed, Argos positions are unbiased and their estimation errors have standard deviations close to 1 km in the case of sea turtles, somewhat smaller for oceanographic buoys [51]. Resulting velocity estimates are thus also unbiased and simple algebra shows that velocities computed using (2) with $\Delta t$ = 1 day have standard deviations on the order of 2 cm/s. They are generally a bit reduced using appropriate position filtering methods, e.g. [52].

Drift velocity estimation errors ${{\varvec{V}}}_{{\varvec{d}}}^{\boldsymbol{^{\prime}}}$ are unfortunately larger and more difficult to characterize. Drift velocity is generally estimated using operational ocean model outputs. Its current-induced component alone (the Eulerian current velocity ${{\varvec{V}}}_{{\varvec{c}}}$) has basin-scale mean errors of the order of 1 cm/s and standard deviations around 10 cm/s [53]. Larger errors are expected at the regional scale. As discussed in the introduction, such errors might be acceptable for adult sea turtle studies but must be reduced and better quantified when estimating the weaker swimming velocities of hatchlings and juveniles.

A model that provides an improved estimation of the drift velocity, including its wind-and wave-induced components, will be presented in Sect. “Drift velocity estimation”. Quantification of the estimation noise can readily be obtained using Eq. (4) with passive drifters for which ${{\varvec{V}}}_{{\varvec{s}}}=0$. In that case, (4) reduces to:

5 $${{\varvec{V}}}_{{\varvec{D}}{\varvec{C}}}={\widehat{{\varvec{V}}}}_{{\varvec{g}}}-{\widehat{{\varvec{V}}}}_{{\varvec{d}}}=\boldsymbol{ }{{{\varvec{V}}}_{{\varvec{d}}}^{\boldsymbol{^{\prime}}}}_{\boldsymbol{ }}-{{\varvec{V}}}_{{\varvec{g}}}^{\boldsymbol{^{\prime}}}$$

The DC-velocity of passive drifters is thus a direct measure of ${{{\varvec{V}}}_{{\varvec{d}}}^{\boldsymbol{^{\prime}}}}_{\boldsymbol{ }}-{{\varvec{V}}}_{{\varvec{g}}}^{\boldsymbol{^{\prime}}}$. Of course, errors affecting the DC-velocities of buoys are not strictly identical to those affecting turtles, but regions where drifters have large model-derived DC-velocities, undoubtedly are regions where turtles’ swimming velocities estimated using (4) also have large errors. More precisely, the statistical distribution of the estimation noise (${{{\varvec{V}}}_{{\varvec{d}}}^{\boldsymbol{^{\prime}}}}_{\boldsymbol{ }}-{{\varvec{V}}}_{{\varvec{g}}}^{\boldsymbol{^{\prime}}}$) is bound to be similar for drifters and for turtles if, in both cases, this noise is generated by the same input errors going through the same transfer function, i.e. the same estimation model. This is already the case for the ${{\varvec{V}}}_{{\varvec{g}}}^{\boldsymbol{^{\prime}}}$ error term which results from Argos positioning errors transformed into velocity over ground estimation errors using the same velocity estimation Eq. (2) for both sea turtles and drifters. To ensure that this is also the case for ${{\varvec{V}}}_{{\varvec{d}}}^{\boldsymbol{^{\prime}}}$, the same drift velocity estimation model will be applied to both drifters and sea turtles (see Sect. “Drift velocity estimation”).

The drift-corrected velocity of passive drifters being a good proxy for the estimation noise affecting sea turtles’ swimming velocity estimates, maps of the mean and standard deviation of this velocity are most helpful. Given the high volume of oceanographic buoy trajectories available from the Global Drifter Program (GDP, https://www.aoml.noaa.gov/global-drifter-program/) high-quality maps can easily be produced for the North Pacific Ocean basin where our turtles are tracked.

Besides such maps, DC-trajectories, an improved version of the current-corrected trajectories proposed by Gaspar et al. [49], also prove to be very useful. A DC-trajectory is defined here as the time integral of the DC-velocity:

6 $${{\varvec{X}}}_{{\varvec{D}}{\varvec{C}}}\left(t\right)={\int }_{{t}_{0}}^{t}{{\varvec{V}}}_{{\varvec{D}}{\varvec{C}}}dt= {\int }_{{t}_{0}}^{t}\left({\widehat{{\varvec{V}}}}_{{\varvec{g}}}-{\widehat{{\varvec{V}}}}_{{\varvec{d}}}\right)dt= {\int }_{{t}_{0}}^{t}{{\varvec{V}}}_{{\varvec{s}}}dt+{\int }_{{t}_{0}}^{t}{({{\varvec{V}}}_{{\varvec{d}}}^{\boldsymbol{^{\prime}}}}_{\boldsymbol{ }}-{{\varvec{V}}}_{{\varvec{g}}}^{\boldsymbol{^{\prime}}}) dt$$

This integral is defined in the ($x,y$) plane, not at the Earth surface, and the initial position ${{\varvec{X}}}_{{\varvec{D}}{\varvec{C}}}\left({t}_{o}\right)$ is (0,0). The position at time $t$ measures the distance traveled since ${t}_{o}$ under the influence of the DC-velocity, in the zonal direction ${x}_{DC}\left(t\right)$ (positive eastwards) and in the meridional direction ${y}_{DC}\left(t\right)$ (positive northwards).

Equation (6) shows that the DC-trajectory of a turtle is the sum of the trajectory generated by its swimming velocity plus a trajectory generated by the estimation noise. For a buoy, the DC-trajectory simply is the trajectory generated by the estimation noise. If, in the same area, turtles DC-trajectories display behaviors not observed in buoys DC-trajectories, then these behaviors can safely be interpreted as signs of the turtles’ swimming activity.

In practice, simple numerical integration is used to estimate DC-trajectories at discrete observation times:

7 $${{\varvec{X}}}_{{\varvec{D}}{\varvec{C}}}\left({t}_{k}\right)= \sum_{i=0}^{k}{{\varvec{V}}}_{{\varvec{D}}{\varvec{C}}}\left({t}_{i}\right)\Delta t$$

DC-trajectories being often convoluted, separate analyses of their zonal ${x}_{DC}\left(t\right)$ and meridional ${y}_{DC}\left(t\right)$ components will prove to be easier and more informative.

### Drift velocity estimation

The dynamics of surface floating objects drifting under the combined effects of currents, winds and waves are complex and their theoretical bases are still a subject of active research, largely motivated by the need to better understand the drift of an ever growing number of marine debris worldwide [54–58]. While theoretical progress is underway, estimates of the drift velocity have been developed for practical use in search and rescue operations [59] and marine pollution monitoring [60, 61]. They take the generic form:

8 $${\widehat{{\varvec{V}}}}_{{\varvec{d}}}= {\widehat{{\varvec{V}}}}_{{\varvec{c}}}+\widehat{{\varvec{L}}}$$

where ${\widehat{{\varvec{V}}}}_{{\varvec{c}}}$ is an estimate of the Eulerian current velocity in the water layer within which the drifter is floating, and $\widehat{{\varvec{L}}}$ is an estimate of the leeway, that is the wind- and wave-induced velocity of the drifter relative to the Eulerian current [62]. Estimates of Eulerian current velocities in different water layers are readily available from operational ocean models, e.g. [63]. Estimation of the leeway is slightly more complex.

The leeway can be expressed as the sum of a wind-induced velocity (${{\varvec{V}}}_{{\varvec{w}}}$) called the windage, and a wave-induced velocity called the Stokes drift (${{\varvec{V}}}_{{\varvec{s}}{\varvec{t}}}$):

9 $${\varvec{L}}={{\varvec{V}}}_{{\varvec{w}}}+{{\varvec{V}}}_{{\varvec{s}}{\varvec{t}}}$$

The Stokes drift ${{\varvec{V}}}_{{\varvec{s}}{\varvec{t}}}$ is a downwave drift velocity induced by the orbital motion that water particles experience under the influence of the surface wave field (see [54] for details). It increases with the wave height. ${{\varvec{V}}}_{{\varvec{s}}{\varvec{t}}}$ is maximum at the surface where it can reach 10 cm/s or more [64]. This is far from negligible compared to Eulerian surface currents which mean values are close to 20 cm/s. ${{\varvec{V}}}_{{\varvec{s}}{\varvec{t}}}$ however decreases rapidly with depth. It is typically halved within the first meter of water and becomes negligible (< < 1 cm/s) at depths between 15 and 20 m [64]. The exact rate of decrease depends on the surface wave field composition, being larger for wind waves than for swell [65].

Windage ${{\varvec{V}}}_{{\varvec{w}}}$ is the velocity induced by the wind drag on the emerged part of the drifting object. It depends on the shape, size and buoyancy of the drifter and increases with the wind speed. The windage is generally decomposed into a downwind and a crosswind component. The crosswind component is small, and usually neglected, in close to radially symmetric objects [62] like oceanographic buoys [66] and in low floating material like sargassum or small marine debris [67, 68]. It also proves to be negligible for surface floating sea-turtle carcasses [69]. In that case, the windage vector is aligned with the wind speed vector.

While the windage and Stokes drift are different effects, they are generally modelled together because, for most practical purposes, only their sum (the leeway) matters and because they are extremely correlated. Indeed, the downwave direction of the Stokes drift is generally close to the downwind direction of the windage and the Stokes drift increases with the windage as the wave height increases with the wind speed. The leeway is thus commonly estimated as a fraction of the wind speed, typically the order of 1% of ${\widehat{{\varvec{W}}}}_{10},$ the wind speed measured at 10 m. However, operational oceanography centers recently made available estimates of the surface Stokes drift ${\widehat{{\varvec{V}}}}_{{\varvec{s}}{\varvec{t}}{\varvec{o}}}$ [70] and it was showed [71] that simulated trajectories of surface drifting buoys are of similar quality when modeling the leeway as a fraction of either ${\widehat{{\varvec{W}}}}_{10}$ or ${\widehat{{\varvec{V}}}}_{{\varvec{s}}{\varvec{t}}{\varvec{o}}}$. For reasons that will be explained later, we select to estimate the leeway as a function of ${\widehat{{\varvec{V}}}}_{{\varvec{s}}{\varvec{t}}{\varvec{o}}}$ so that the drift velocity of both oceanographic drifters and sea turtles will be modeled under the simple form:

10 $${\widehat{{\varvec{V}}}}_{{\varvec{d}}}={\widehat{{\varvec{V}}}}_{{\varvec{c}}}+\boldsymbol{ }\gamma \boldsymbol{ }{\widehat{{\varvec{V}}}}_{{\varvec{s}}{\varvec{t}}{\varvec{o}}}$$

To close this model, we only have to determine the value of the $\gamma$ parameter and choose the ocean model layer from which ${\widehat{{\varvec{V}}}}_{{\varvec{c}}}$ must be extracted.

#### The case of surface buoys

For surface buoys, the obvious choice for ${\widehat{{\varvec{V}}}}_{{\varvec{c}}}$ is the current velocity within the upper model layer (${\widehat{{\varvec{V}}}}_{{\varvec{c}}{\varvec{o}}}$). Furthermore, optimal estimation of $\gamma$ can be directly achieved from observations. Indeed, since ${{\varvec{V}}}_{{\varvec{s}}}=0$, the velocity over ground is the drift velocity so that an estimate of the leeway is readily obtained under the form:

11 $${\widehat{{\varvec{L}}}=\boldsymbol{ }\widehat{{\varvec{V}}}}_{{\varvec{g}}}-{\widehat{{\varvec{V}}}}_{{\varvec{c}}{\varvec{o}}}$$

The value of $\gamma$ that provides the best least-square fit to this observed leeway is then simply obtained from the 2-D regression:

12 $${\widehat{{\varvec{V}}}}_{{\varvec{g}}}-{\widehat{{\varvec{V}}}}_{{\varvec{c}}{\varvec{o}}}=\boldsymbol{ }\gamma {\widehat{{\varvec{V}}}}_{{\varvec{s}}{\varvec{t}}{\varvec{o}}}+{\varvec{\mu}} + \varvec{\epsilon}$$

where ${\varvec{\mu}}$ is the intercept and ${\varvec{\epsilon}}$ the regression residual.

#### The case of sea turtles

The impact of the leeway on sea turtle dispersal is rarely mentioned (for exceptions, see [44, 72]). It has, so far, always been neglected in sea turtle movement analyses [10, 49, 50] and in simulations of juvenile sea turtles dispersal [12, 15, 31, 73]. Neglecting the leeway may be acceptable for individuals that spend little time at or close to the surface as they are rarely subject to windage and mostly experience a reduced or negligible Stokes drift. This is not the case for juvenile loggerheads that spend a large fraction of their time at the sea surface or within the first few meters of water [9, 23, 74]. More precisely, dive data from several individuals included in the tracking data set used here reveals that these juveniles spend about 20% of their time at the surface or within the first meter of water, more than 85% of their time above 15 m and over 95% of their time above 20 m [46]. Their drift velocity thus clearly has a leeway component which will be modeled using (10). The choice to parameterize the leeway as a fraction of ${\boldsymbol{ }\widehat{{\varvec{V}}}}_{{\varvec{s}}{\varvec{t}}{\varvec{o}}}$ rather than ${\widehat{{\varvec{W}}}}_{10}$ is justified here as dive data show that our tracked individuals are almost always under the influence of the Stokes drift while windage only affects them for about 20% of their time.

Final closure of the sea turtle’s version of the drift velocity model (10), requires that we estimate the value of $\gamma$ for juvenile loggerheads and choose the ocean model layer from which ${\widehat{{\varvec{V}}}}_{{\varvec{c}}}$ is extracted. In theory, the layer selection must continuously follow the diving depth of the tracked individual [75]. In our case, dive data show [46] that they are almost permanently within 20 m from the surface, that is generally within the upper oceanic mixed layer where turbulence maintains Eulerian current velocities close to their surface value. We will thus assume ${\widehat{{\varvec{V}}}}_{{\varvec{c}}}={\widehat{{\varvec{V}}}}_{{\varvec{c}}{\varvec{o}}}$. Estimation of $\gamma$ will then be performed using regression (12), just like we did for surface buoys. For sea turtles however, the velocity over ground is no longer strictly equal to the drift velocity but includes the swimming velocity. Part of the swimming velocity could thus be absorbed in the parameterized leeway if ${{\varvec{V}}}_{{\varvec{s}}}$ is correlated with ${\widehat{{\varvec{V}}}}_{{\varvec{s}}{\varvec{t}}{\varvec{o}}}$. A causal correlation can hardly be envisioned but an empirical correlation is always possible, especially in small data sets. This is not the case here as the sea turtle tracking data set we will use is unusually large (over 80,000 velocity measurements) and covers a wide range of swimming activities. Regression results will confirm that the $\gamma$ estimate for sea turtles is consistent with the known physics of the Stokes drift and the estimate obtained for surface buoys.

### Sea turtle data

#### Tracking data

**Fig. 1:**
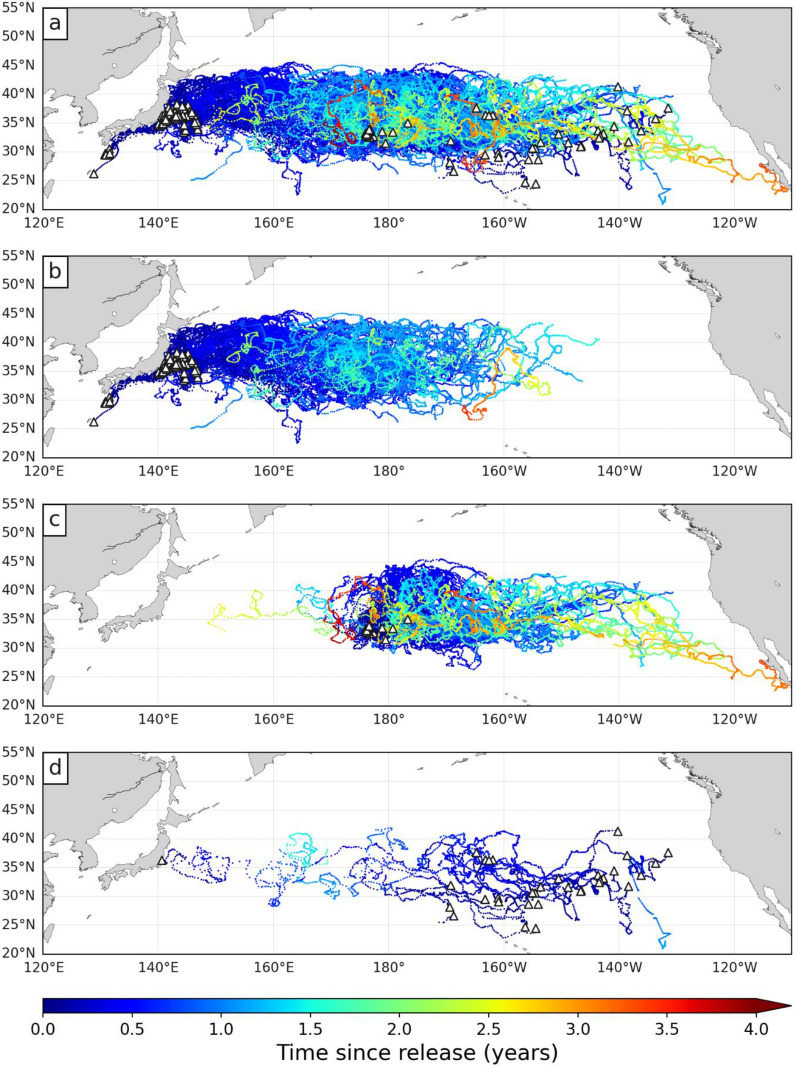
Trajectories of all juvenile loggerhead turtles in the NPLT data set. Small triangles indicate turtles’ release positions. **a** All turtles (n = 232), **b** raised turtles released off Japan (n = 124), **c** raised turtles released in the CNP (n = 74) and **d** wild turtles released near capture positions (n = 34). The color along each trajectory evolves as a function of the time since release

This paper exploits the trajectories of 232 juvenile loggerheads released and satellite-tracked in the North Pacific Ocean under the NOAA PIFSC Marine Turtle Research Program between 1997 and 2013 (Fig. 1a). This includes the 231 trajectories previously described and analysed by Briscoe et al. [43] plus one 22-month long trajectory omitted from that analysis. This trajectory belongs to a turtle captive-reared in Japan and released in 2005 with a straight carapace length (SCL) of 31.1 cm. This entire data set will be referred to as the NP Loggerhead Tracking (NPLT) data set.

Most tracked turtles (n = 198) were hatched and reared in the Port of Nagoya Public Aquarium. The others were incidentally captured (n = 34). Reared individuals were released between 2003 and 2011 either in the Western North Pacific (WNP) off the Japanese coast in the Kuroshio current (n = 124) (Fig. 1b) or near the International Date Line, in the Central North Pacific (CNP) (n = 74) (Fig. 1c). Individuals from these two groups will be referred to as the WNP and CNP turtles respectively. Captured individuals (n = 34) were released between 1997 and 2003 and will be referred to as the wild turtles. Their release positions are broadly dispersed, mostly in the central and eastern North Pacific basin. A single individual was released close to the Japanese coast (Fig. 1d). All turtles were equipped with Argos-linked transmitters. Their raw locations were filtered and resampled at regular 24-h intervals (12:00 UTC every day) using the Bayesian State Space Switching Model of Jonsen et al. [52] as described by Briscoe et al. [43]. A total of 86,105 daily positions were obtained in this way, indicating a mean individual tracking duration slightly above one year (range: 4 to 1434 days) The mean tracking duration of wild turtles (156 days) is shorter than that of captive-reared turtles (408 days), likely because of a higher post-release mortality [76].

#### Size and growth

**Fig. 2:**
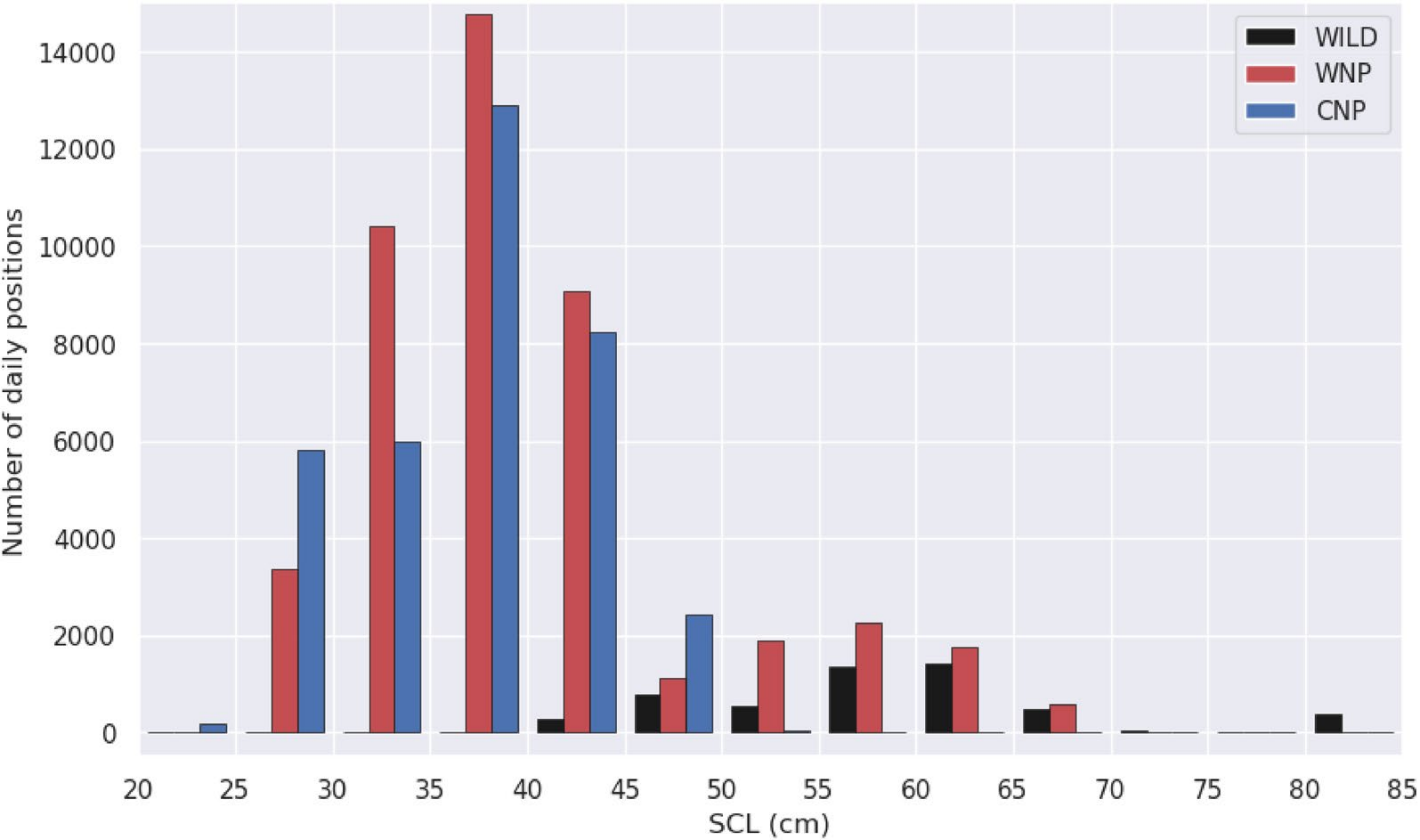
Histogram of estimated individual sizes at each recorded daily position for wild (black), WNP (red) and CNP (blue) turtles

A main goal of this paper is to examine how the swimming behavior evolves with size. Tracked turtles have sizes ranging from 24.4 to 83 SCL at the time of release [43]. Significant growth is expected during the tracking period, especially in the youngest individuals tracked for several years. Therefore, to keep up with sizes during the complete tracking period, we assume that the SCL of each individual follows a classical von Bertalanffy growth curve starting from ${L}_{o}$, its value measured on ${t}_{0}$, the date of release:

13 $$L\left(t\right)={L}_{\infty }-\left[{L}_{\infty }-{L}_{o}\right]{e}^{-K\left(t-{t}_{0}\right)}$$

where $L\left(t\right)$ is the SCL at any time $t$ > ${t}_{0}$, ${L}_{\infty }\text{ the asymptotic length}$ and $K$ the growth coefficient. Conservative values of these growth parameters (${L}_{\infty }$=100.06 cm and $K$ = 0.066/year) are taken from the Ramirez et al. [77] global synthesis of loggerhead growth data. The size of every tracked individual is then estimated on a daily basis and this information is added to daily position records. The histogram of the estimated sizes is shown in Fig. 2, separating the WNP, CNP and wild turtle groups. The peak of the SCL distribution is close to 40 cm. Individuals smaller than this size are exclusively captive-reared turtles (WNP and CNP). Large individuals (SCL > 50 cm) are exclusively WNP or wild turtles. The maximum estimated individual SCL increase over the tracking period is 14.6 cm (mean = 4 ± 2.8 cm).

### Surface buoy data

Trajectories of surface buoys from the Global Drifter Program, processed and made available by the Copernicus Marine Service (CMEMS, https://marine.copernicus.eu/access-data) are used here. These buoys are designed to follow Eulerian currents at a nominal depth of 15 m with minimum perturbation from the windage and Stokes drift. They consist of a small spherical surface float (diameter ~ 35 cm) attached to a drogue centered at 15-m depth, but the drogue attachment sometimes breaks. When this happens, the surface float starts following the current within the first meter of water and becomes clearly influenced by windage and Stokes drift. This change in the drift behavior is systematically detected [78, 79] so that all buoy positions in the CMEMS data base come with a “drogue status flag” indicating whether they belong to a drogued or undrogued float. Only data from undrogued buoys will be used here since, like juvenile loggerheads (but unlike drogued buoys), their drift velocities have a leeway component whose estimation error we aim to quantify.

**Fig. 3:**
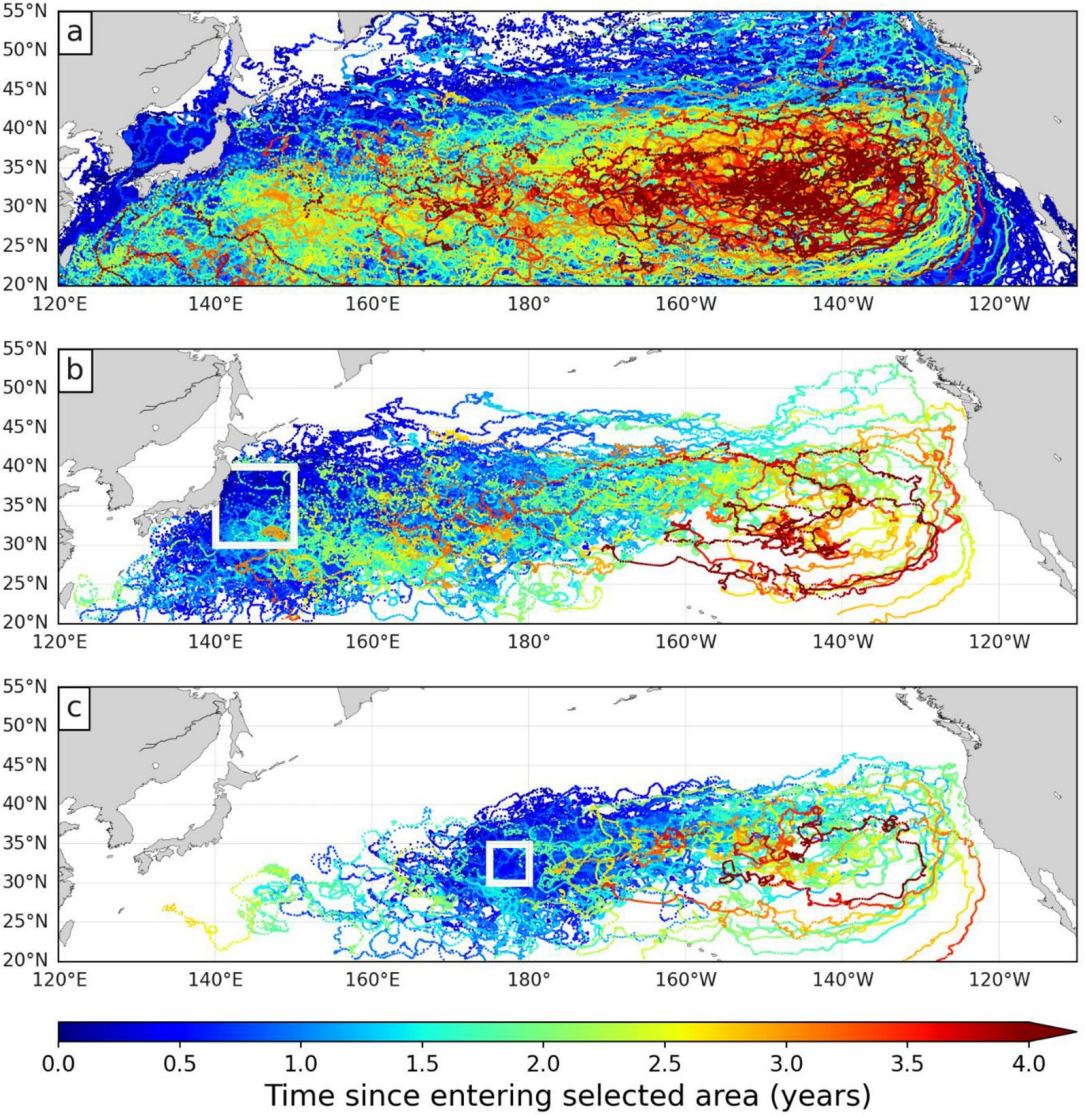
Trajectories of undrogued GDP surface buoys tracked in the NP Ocean between 1997 and 2013. **a** All buoys (n = 1695), **b** buoys passing off Japan through the area delineated by a white square (n = 269) and **c** buoys passing in the CNP through the area delineated by a white square (n = 138). The color along each trajectory evolves as a function of the time since entering the selected area

We therefore extracted, from the CMEMS data base, the trajectories of all undrogued GDP drifters circulating in the NP basin [20–55°N; 120°E-110°W] between 01/01/1997 and 31/12/2013 (Fig. 3a). This largely covers the NPLT tracking area and period. GDP drifter positions are provided at regular 6-h intervals but only one position per day (at 12:00 UTC) was retained so that the temporal sampling of the buoy and turtle trajectories is identical. The so-obtained NP buoy data set contains nearly half a million daily positions, representing the trajectories of 1695 undrogued drifters tracked for a mean period of 310 days (range: 2 to 3109 days) within the selected area and period. Note that they end up concentrating in the great garbage patch area (Fig. 3a).

Out of this complete NP data set, we extracted a WNP and a CNP subset. The first one contains the trajectories of all buoys passing along Japan, in the area [30–40°N; 140–150°E] (Fig. 3b) where most WNP turtles were released (n = 107). The others (n = 17) were released further south but were then rapidly entrained into this area by the Kuroshio current. The second subset contains the trajectories of all buoys passing through the area [30–35°N; 175–180°E] where all CNP turtles were released (Fig. 3c). Each trajectory in these subsets begins when a drifter enters the selected area and stops when it exits the NP basin, or when the final date of 31/12/2013 is reached. The WNP buoys subset contains 269 trajectories with a mean duration of 402 days (range: 2 to 2061 days). The CNP buoys subset includes 138 trajectories with a mean duration of 447 days (range: 6 to 1702 days).

### Ocean and wave model data

As for the buoy data, we extracted surface Eulerian current and Stokes drift data from the CMEMS data base. The selected data cover the same NP area [20–55°N; 120°E-110°W] for the period 1997 to 2013. Eulerian surface current vectors ${\widehat{{\varvec{V}}}}_{{\varvec{c}}{\varvec{o}}}$ are outputs of the eddy-resolving GLORYS12 reanalysis of the World Ocean circulation performed with the NEMO model [63]. Daily averaged (00:00 to 24:00 UTC) currents are provided on a regular 1/12° horizontal grid. Data from the first model layer (0 to 1 m depth) are used.

**Fig. 4:**
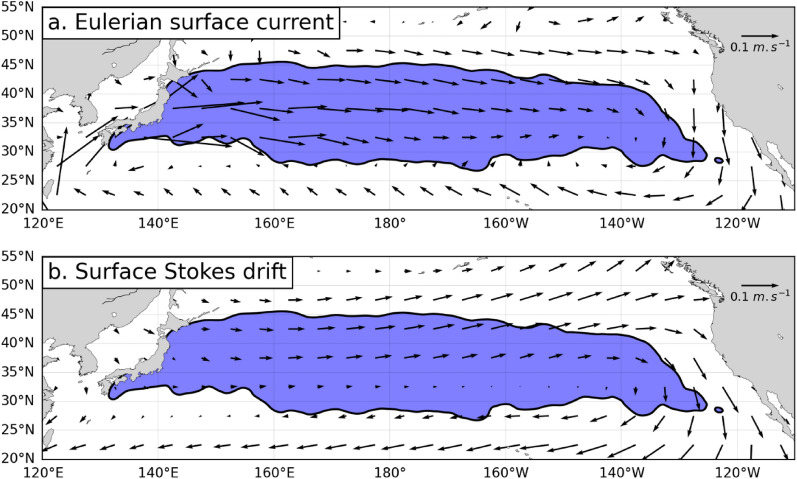
Maps of **a** Eulerian surface currents from the GLORYS12 reanalysis and **b** surface Stokes drift from the WAVERYS reanalysis. Data are averaged over 5° × 5° boxes during the period 01/01/1997 to 31/12/2013. The area shaded in blue delineates the area with the highest density of recorded turtle positions (97% of all turtle positions are found inside this area)

Surface Stokes drift vectors ${\widehat{{\varvec{V}}}}_{{\varvec{s}}{\varvec{t}}{\varvec{o}}}$ are a product of the WAVERYS reanalysis of the global wave field performed with the MFWAM model [70] which takes into account the effect of the GLORYS12 currents on the wave field. Three-hourly mean Stokes drift vectors are provided on a regular 1/5° horizontal grid. We averaged them to obtain daily mean (0:00 to 24:00 UTC) Stokes drift vectors which temporal sampling matches that of the Eulerian currents. Maps of the Eulerian surface currents and Stokes drift averaged over 5° × 5° boxes during the whole period 1997 to 2013 are shown in Fig. 4.

### Matching tracking and current data

The Eulerian currents and Stokes drift being available as daily mean values, they must be matched with velocities over ground computed over the same 24-h period, that is the movement of buoys or turtles, between 0:00 UTC and 24:00 UTC. To do so, buoy and turtle positions at 0:00 UTC were estimated by linear interpolation between the two adjacent positions at 12:00 UTC. The daily mean velocity over ground was then computed using (2) and recorded together with the daily positions at 12:00 UTC. Estimates of the daily mean Eulerian current and Stokes drift at that position were then obtained by bilinear interpolation in space of the gridded modeled data of the day.

This data processing procedure finally yields two similarly processed data sets containing for the first one 86,105 records of turtle positions and speeds and, for the second one, 485,574 records of surface buoy positions and speeds. Each record contains:

[Individual ID, group ID, SCL (turtle only), date, position at 12:00 UTC, ${\widehat{{\varvec{V}}}}_{{\varvec{g}}},\boldsymbol{ }{\boldsymbol{ }\widehat{{\varvec{V}}}}_{{\varvec{c}}{\varvec{o}}},\boldsymbol{ }{\boldsymbol{ }\widehat{{\varvec{V}}}}_{{\varvec{s}}{\varvec{t}}{\varvec{o}}}$]

The group ID indicates whether a turtle belongs to the WNP, CNP or wild group and whether a buoy belongs to the WNP or CNP subset.

## Results

### Drift velocity estimates

Regression (12) performed with the complete NP buoy and NPLT data sets yields the following estimates of the drift velocities:

14a $$\text{For surface buoys}:{\widehat{{\varvec{V}}}}_{{\varvec{d}}}= {\widehat{{\varvec{V}}}}_{{\varvec{c}}{\varvec{o}}}+\boldsymbol{ }0.88\boldsymbol{ }{\widehat{{\varvec{V}}}}_{{\varvec{s}}{\varvec{t}}{\varvec{o}}}$$

14b $$\text{For sea turtles}: {\widehat{{\varvec{V}}}}_{{\varvec{d}}}= {\widehat{{\varvec{V}}}}_{{\varvec{c}}{\varvec{o}}}+\boldsymbol{ }0.28\boldsymbol{ }{\widehat{{\varvec{V}}}}_{{\varvec{s}}{\varvec{t}}{\varvec{o}}}$$

For buoys, a value of $\gamma$ close to, but slightly below, one is in line with expectations for small surface floats drifting within the first 20 or 30 cm of water: their minimal windage is not sufficient to compensate for the rapid decrease with depth of the Stokes drift. For sea turtles, a value of $\gamma$ significantly smaller than for surface drifters was also expected as juvenile loggerheads only spend a fraction (~ 20%) of their time under the influence of windage and dive mostly between 1 and 15 m [46], that is in a range of depths where the Stokes drift is much weaker than at the surface. The norm of the mean leeway correction $\Vert 0.28\boldsymbol{ }{\widehat{{\varvec{V}}}}_{{\varvec{s}}{\varvec{t}}{\varvec{o}}}\Vert$ along the loggerhead trajectories is only 2.4 ± 1.4 cm/s, an order of magnitude smaller than the main current-induced drift component (21.2 ± 20.9 cm/s). Such a small correction could be neglected in short-term studies but must be considered when dealing with long trajectories as a 1 cm/s mean error on the drift velocity can shift a DC- trajectory by more than 300 km over a year.

**Fig. 5:**
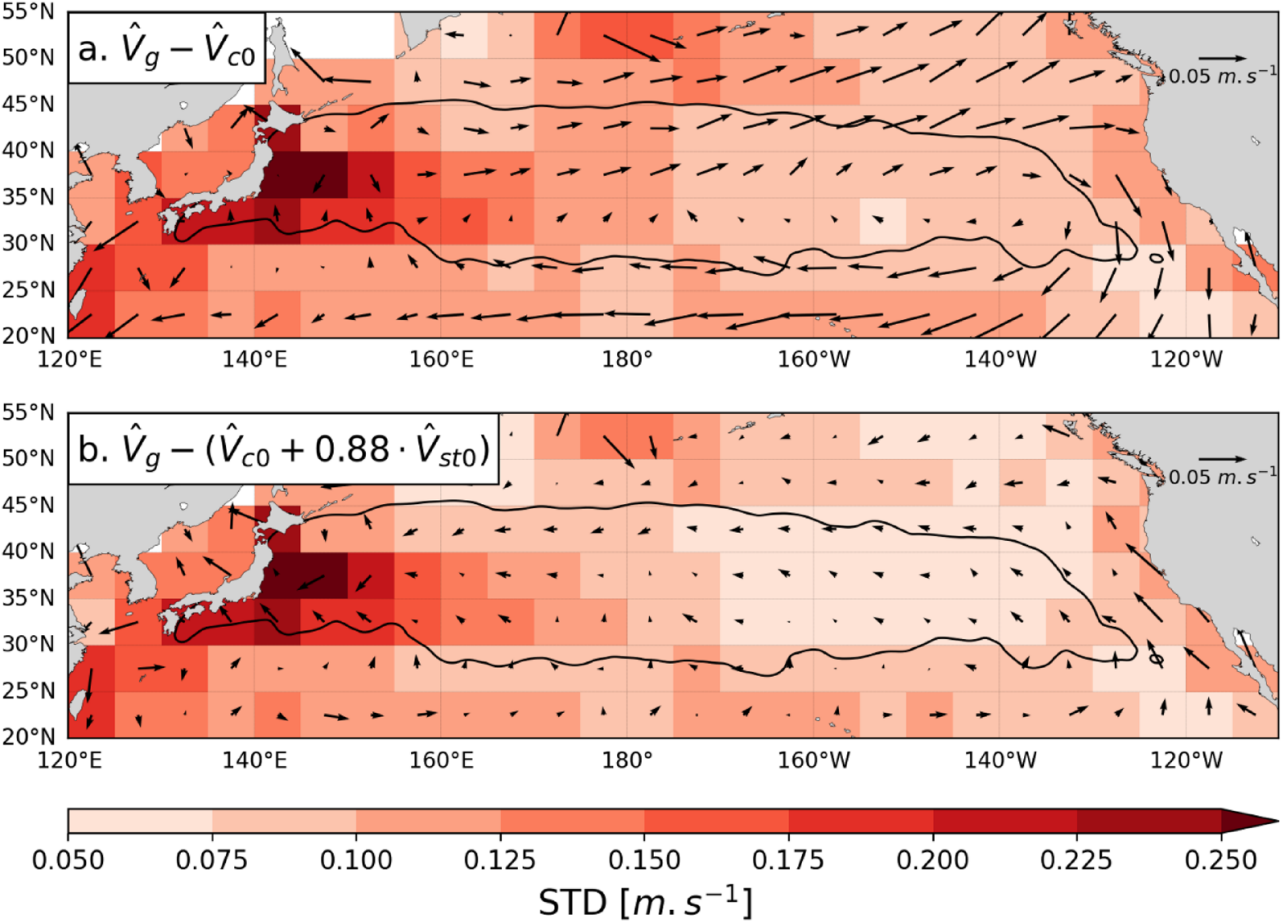
Maps of surface buoys’ mean DC-velocities assuming **a** ${\widehat{{\varvec{V}}}}_{{\varvec{d}}}= {\widehat{{\varvec{V}}}}_{{\varvec{c}}{\varvec{o}}}$ or **b** ${\widehat{{\varvec{V}}}}_{{\varvec{d}}}= {\widehat{{\varvec{V}}}}_{{\varvec{c}}{\varvec{o}}}+\boldsymbol{ }0.88\boldsymbol{ }{\widehat{{\varvec{V}}}}_{{\varvec{s}}{\varvec{t}}{\varvec{o}}}$. Mean DC-velocity vectors and standard deviations of their norm are computed over 5° × 5° areas. The background color in each area is a function of the standard deviation

It is especially interesting to measure the accuracy gain obtained when adding the leeway correction to drift velocity estimates. To this aim, we computed the DC-velocities of all NP surface buoys assuming either ${\widehat{{\varvec{V}}}}_{{\varvec{d}}}= {\widehat{{\varvec{V}}}}_{{\varvec{c}}{\varvec{o}}}$ or ${\widehat{{\varvec{V}}}}_{{\varvec{d}}}= {\widehat{{\varvec{V}}}}_{{\varvec{c}}{\varvec{o}}}+\boldsymbol{ }0.88\boldsymbol{ }{\widehat{{\varvec{V}}}}_{{\varvec{s}}{\varvec{t}}{\varvec{o}}}$. The mean and standard deviation of these DC-velocities, binned into regular 5° × 5° boxes are displayed in Fig. 5. When the leeway correction is omitted (${\widehat{{\varvec{V}}}}_{{\varvec{d}}}= {\widehat{{\varvec{V}}}}_{{\varvec{c}}{\varvec{o}}}$), mean DC-velocities commonly reach or exceed 5 cm/s and display a clear clockwise pattern, resembling that of the Stokes drift (see Fig. 4b). Adding a leeway correction (Fig. 5b) largely eliminates this error pattern and reduces drift estimation errors. The reduction of the mean drift velocity estimation error, and of its standard deviation, is most visible in the central and eastern Pacific. Mean errors, and standard deviations are also somewhat reduced in the western Pacific but remain relatively large, especially in the Kuroshio area near the Japanese coast. This was expected as, in this dynamically active area, occasional mispositioning of mesoscale features and insufficiently resolved sub-mesoscale activity can induce large errors in modeled Eulerian current velocities which cannot be corrected for by the Stokes drift.

### Surface buoys DC-trajectories

**Fig. 6:**
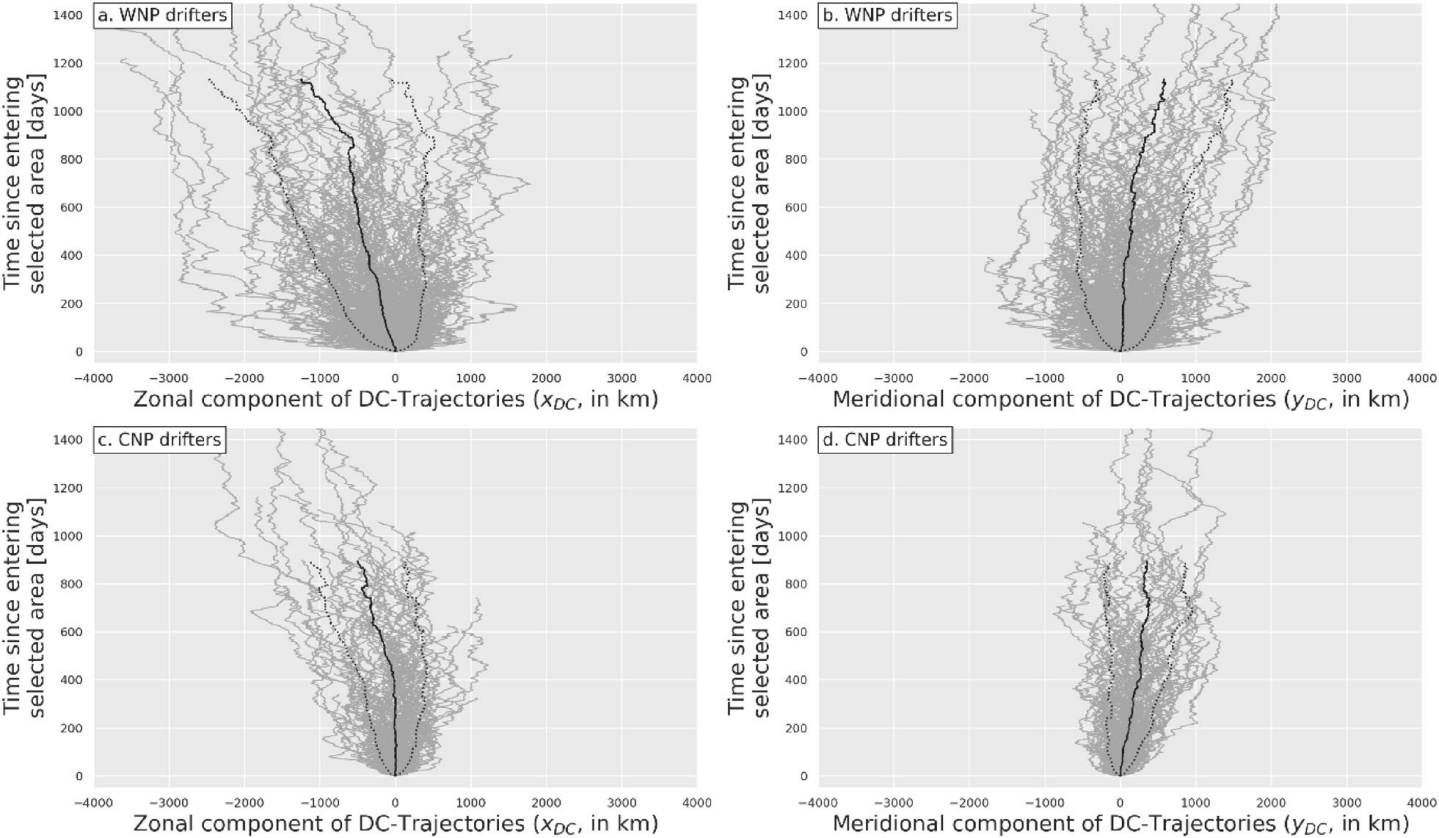
Zonal and meridional components [${x}_{DC}\left(t\right)$, ${y}_{DC}\left(t\right) ]$ of DC-trajectories of WNP buoys (**a**, **b**) and CNP buoys (**c**, **d**). In each figure, the solid black line is the ensemble-mean distance ${\overline{x} }_{DC}\left(t\right)\text{ or}$ ${\overline{y} }_{DC}\left(t\right)$ and the dotted lines are this mean ± one standard deviation. These lines are discontinued when the number of trajectories drops below 20

DC-trajectories were computed for all surface buoys and tracked turtles using drift velocity estimates (14a) and (14b), respectively. The zonal and meridional components [${x}_{DC}\left(t\right)$, ${y}_{DC}\left(t\right) ]$ of the DC-trajectories of all WNP and CNP surface buoys are shown in Fig. 6. They are plotted over a maximum period of 1434 days which corresponds to the longest sea turtle tracking period. Both zonal and meridional DC-trajectories feature similar erratic movements generating a crudely conic dispersal pattern with small, but non-zero, mean displacements $[{\overline{x} }_{DC}\left(t\right),$ ${\overline{y} }_{DC}\left(t\right)]$ (solid black line) and standard deviations (dotted black lines) increasing with time. No conspicuous organized movements are visible. The mean zonal DC-displacement ${\overline{x} }_{DC}\left(t\right)$ of both WNP and CNP buoys is slightly westwards reaching between 376 km (for CNP buoys) and 615 km (for WNP buoys) after 800 tracking days, indicative of a mean westward DC-velocity between 0.5 and 0.9 cm/s. This is consistent with the magnitude and direction of the mean drift velocity estimation errors displayed in Fig. 5b. The mean meridional DC-displacement is smaller, close to 300 km after 800 days, for both WNP and CNP buoys, also in agreement with Fig. 5b which shows that the meridional component of the drift velocity estimation error is generally smaller than the zonal component.

The DC-trajectories of the WNP buoys disperse more rapidly than those of the CNP buoys, both in the zonal and meridional direction. This is especially noticeable at the beginning of the DC-trajectories. One hundred days after release, the standard deviation of [${x}_{DC}\left(t\right)$, ${y}_{DC}\left(t\right) ]$ is [421 km, 361 km] for WNP buoys but only [219 km, 202 km] for CNP buoys. This is consistent with the observation that the standard deviation of the drift velocity estimation error is maximum close to Japan and steadily decreases as one moves towards CNP (Fig. 5b).

### Sea turtles DC-trajectories

**Fig. 7:**
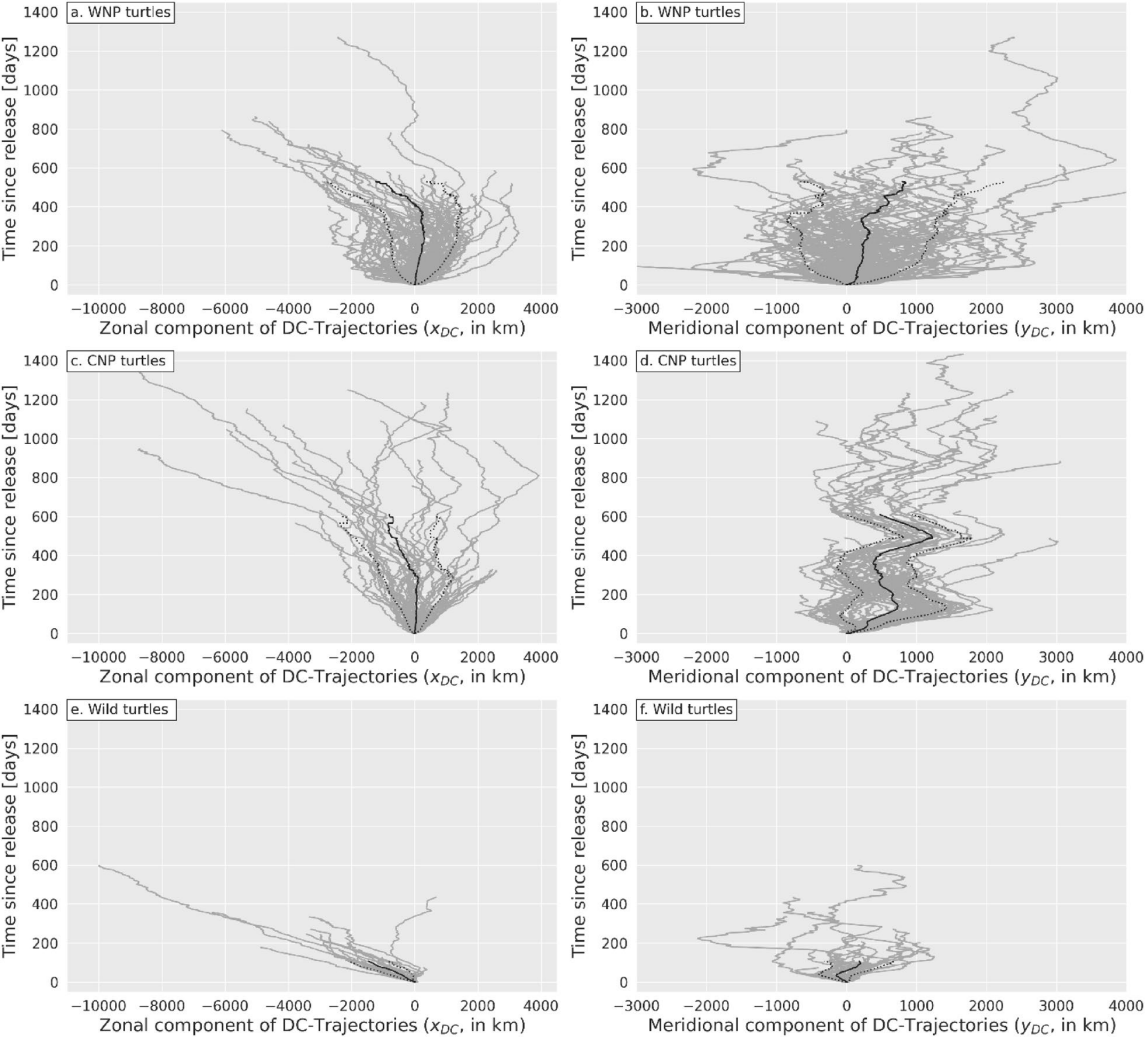
Same as Fig. 6 but for of WNP (**a**, **b**), CNP (**c**, **d**) and wild turtles (**e**, **f**)

The zonal and meridional components of loggerheads’ DC-trajectories (Fig. 7) clearly differ from those of passive drifters thereby demonstrating that these trajectories are not exclusively generated by drift velocity estimation errors but also contain a detectable swimming velocity signal.

#### Zonal component

The difference is clearest in the zonal component of wild turtles’ DC-trajectories (Fig. 7e). In all but one of these trajectories, wild turtles display consistently westwards movements during most of their tracking period, the longest one reaching 10,000 km in 597 days. Estimated zonal DC-velocities are largely negative with a mean close to − 14 cm/s, that is more than 10 times the mean drift velocity estimation error derived from drifter’s DC-trajectories. Such large negative values can only be due to a strong westward swimming activity which allows most wild turtles to move westwards over ground (Fig. 1d) despite the prevailing currents and Stokes drift that push them eastwards (Fig. 4).

Initially, the zonal DC-trajectories of the WNP and CNP turtles (Fig. 7a, c) are more like those of surface buoys: they disperse both eastwards and westwards with no preferred direction and, accordingly, small mean movements. Dispersal however increases more rapidly with time than is the case with passive drifters suggesting that some random swimming activity, possibly linked to foraging, is present. One hundred days after release, the standard deviation of ${x}_{DC}\left(t\right)$ reaches 747 km for WNP turtles and 415 km for CNP turtles, almost double that of surface buoys. Later in the tracking period, markedly westwards DC-trajectory segments, similar to those of wild turtles, appear in a number of WNP and CNP turtles. As a consequence, mean zonal DC-displacement ${\overline{x} }_{DC}\left(t\right)$ become more and more negative. This suggests that after a period of erratic, nearly zero-mean, zonal displacements some turtles change behavior and start swimming consistently westwards until the end of their tracking period, just like most wild turtles do from the beginning of their tracking period.

#### Meridional component

**Fig. 8:**
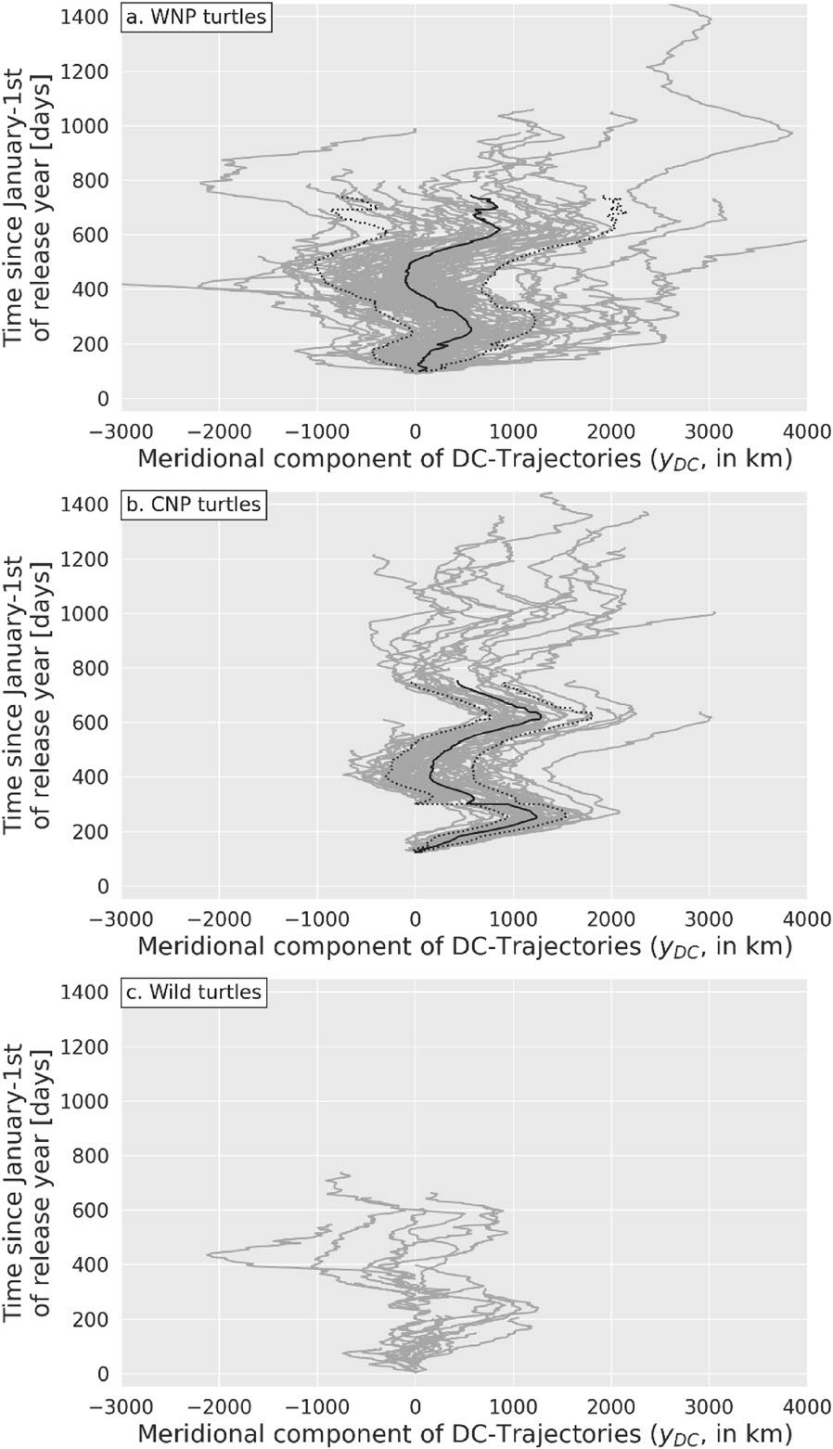
Meridional component ${y}_{DC}\left(t\right)$ of DC-trajectories of **a** WNP **b** CNP and wild **c** turtles plotted as a function of time, the time origin being January 1 st of the year of release. The solid black line is the ensemble-mean distance ${\overline{y} }_{DC}\left(t\right)$ and the dotted lines are this mean ± one standard deviation. These lines are discontinued when the number of trajectories drops below 20

At first glance, the dispersal patterns in meridional DC-trajectories of all turtle groups (Fig. 7b,d,f) are more puzzling but one quickly realizes that large periodic oscillations are present, most visibly in CNP turtles (Fig. 7d). These oscillations reveal the presence of large seasonal migrations, but they are difficult to detect in figures where the origin of the y-axis is the individually-variable release date. Large synchronous seasonal DC-movements become far more visible in all turtle groups in Fig. 8, where the origin of the y-axis is selected to be January 1 st of the year of release.

Such large seasonal oscillations (with amplitudes approaching 1000 km) are clearly absent from passive drifters’ DC-trajectories. They can thus only be explained by a marked meridional swimming activity. This seasonal swimming activity had previously been detected in subsamples of the NPLT data set [8, 40] in which juvenile loggerheads were observed to move northwards during spring and summer and southwards during fall and winter. In doing so, they follow the seasonal meridional movements of the NP Transition Zone Chlorophyll Front (TZCF), a favorable developmental habitat characterized by surface water temperatures around 18 °C and Chlorophyll-a concentrations around 0.2 mg/m^3^ [41, 42, 80, 81].

### Large-scale long-term swimming activities in sea turtles

Visual inspection reveals clear large-scale differences between turtles and buoys DC-trajectories. These differences are indicative of active seasonal meridional movements with amplitudes reaching 1000 km and consistent westwards movements over several months and hundreds or thousands of kilometers These zonal and meridional movements are further investigated below.

#### Zonal swimming activity

Long, markedly westwards, trajectory segments are visible in the zonal DC-trajectories of most wild turtles and some WNP/CNP turtles (Figs. 7 a,c,e). They are associated with steady, largely negative, zonal DC-velocities which persist until the end of these trajectories. In the latitude range where they take place (between 30 and 45°N), these westward swimming movements progressively lead juveniles back towards Japan, their natal island. These segments will accordingly be called homing segments (denoted H segments). The term “homing” is used here in a broad sense, to refer to movements undertaken to reach a known, spatially, restricted area [82]. True natal homing is not envisioned as the tracked individuals are generally too small to have reached sexual maturity and precisely target their natal area. Westbound turtles more likely broadly target the Japanese waters where late stage juveniles, are known to recruit [83]. As none of the westbound turtles reaches Japan before its tag stops transmitting (all are more than 800 km east of Japan when this happens), one could also hypothesize that, instead of the Japanese coastline, juveniles target the rich pelagic foraging grounds found in the western Pacific, in particular in the Kuroshio Extension Bifurcation Region (KEBR) [40]. They might do so to refuel before moving eastwards again through the poorer waters of the Eastern Pacific basin [43]. However, to be targeted, the position of these foraging areas must be known. This likely is the case for wild and WNP turtles that previously crossed these areas after leaving Japan but certainly not for the turtles that were directly released in the CNP. This knowledge would thus need to be innate. While this cannot be excluded, the principle of parsimony leads us to adopt the simpler assumption that individuals (broadly) target Japan, an area they sure all know.

Outside of these H segments, the turtles’ zonal DC-trajectories essentially look like those of passive drifters. They have no clearly preferred directions and are characterized by weak, positive or negative, mean zonal DC-velocities. Such segments will thus be called drifting segments (denoted D segments). This name does not imply that tracked individuals are purely passive within D segments. In fact, they are most probably active as zonal dispersal increases faster during D segments than it does in passive drifter trajectories. This suggests that random movements, often indicative of foraging activities, contribute to increased diffusivity along such segments.

**Fig. 9:**
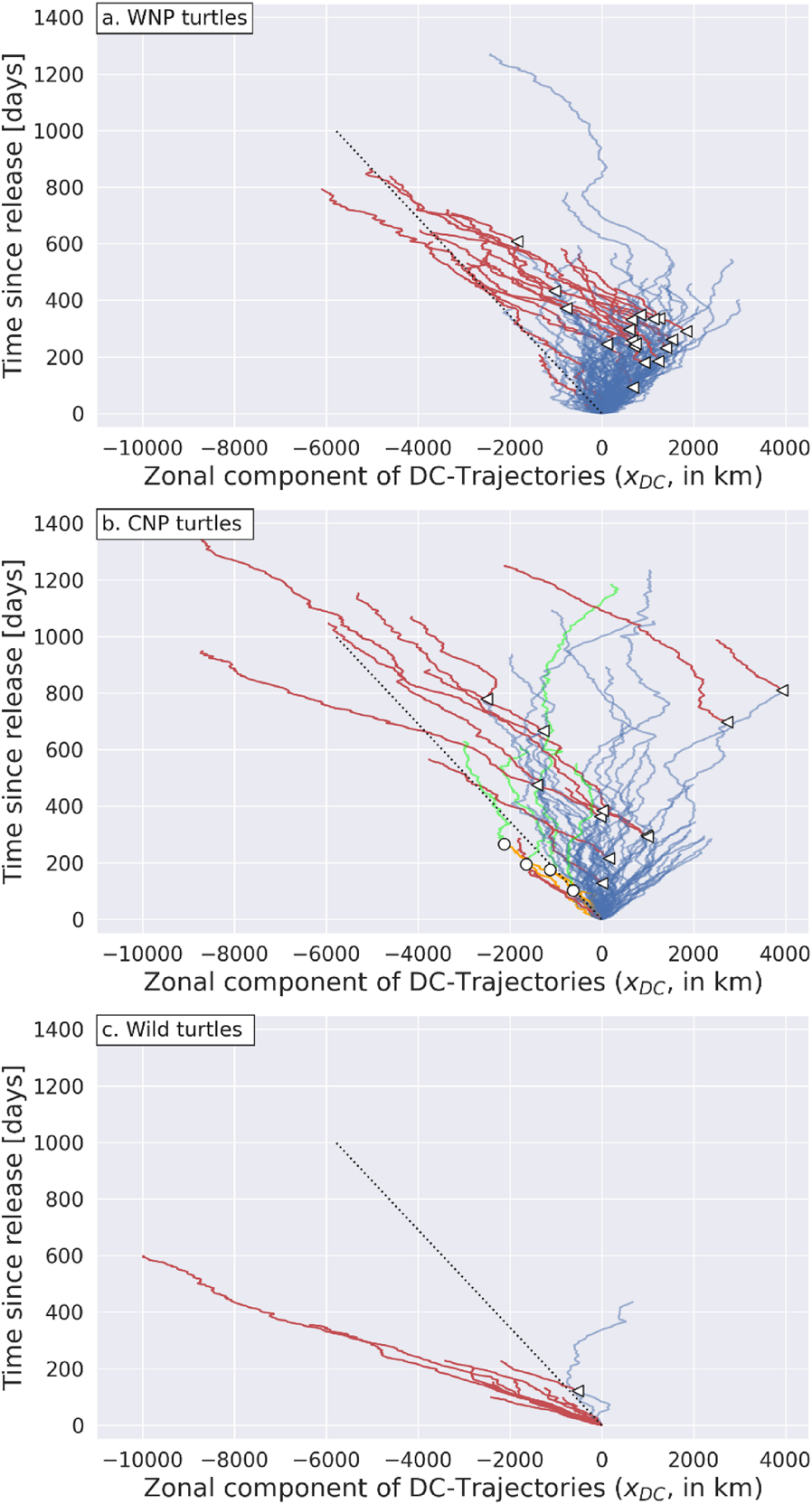
Segmented zonal DC-trajectories ${x}_{DC}\left(t\right)$ of the **a** WNP, **b** CNP and **c** wild turtles. DH breakpoints appear as small triangles, HD breakpoints as small circles. Discarded U segments are not plotted. Trajectories in which one segment has been discarded are plotted as if the tracked individual had been released at the initial position of this valid segment. D segments are in blue and H segments are in red except for the 4 turtles with a HD-trajectory: their initial H segment is in orange and their (second) D segment is in green. In all panels, the dotted black straight line has a slope corresponding to the threshold value ${u}_{DC}$ = −6,5 cm/s

The next step is to progress from visual to automatic identification of the D and H segments. This amounts to automatically detecting when tracked individuals switch from having a close-to-zero zonal mean DC-velocity (D segment) to a markedly negative one (H segment). The method used to perform this type of segmentation, or breakpoint detection, is fully described in Additional File 1. This method systematically identifies 2 segments in any trajectory and then classifies them as D, H or U (unreliable) segments. A segment with a mean zonal DC-velocity $\overline{{u }_{DC}}$ < − 6.5 cm/s is classified H. A segment with $\overline{{u }_{DC}}$ > − 6.5 cm/s is classified D. Too short and/or inhomogeneous segments are classified as unreliable and discarded from further analysis. This concerns segments shorter than 90 days and those in which the standard deviation of ${u}_{DC}$ exceeds 0.25 m/s. Most discarded segments (22 complete trajectories and 146 segments) are short. Analyzing only segments or trajectories longer than 90 days is appropriate here as we only seek to identify long-lasting swimming behaviors, typically at seasonal or longer time scales. Only 22 segments are discarded for being inhomogeneous. Unsurprisingly, they are all located near the Japanese coast where DC-velocities have a large estimation noise. Altogether, discarded segments only represent 12% of the NPLT data set. The remaining 88%, that is 75,461 daily positions and velocity records, belong to either D (70%) or H segments (18%). Some of the subsequent data analyses will be separately carried out on either the ensemble of valid D segments (the D data set) or the ensemble of valid H segments (the H data set). In terms of trajectories, the final result of the segmentation/classification process is detailed in Table 1 and displayed in Fig. 9.

**Table 1:** Final trajectory classification

Trajectory classification	n	Comment
Unreliable (U, discarded)	43	Trajectories < 90 days (n = 22) or with 2 U segments (n = 21)
Drifting only (D)	138	2 D segments (n = 28) or 1 D + 1 U segment (n = 110)
Homing only (H)	17	2 H segments (n = 1) or 1H + 1 U segment (n = 16)
Drifting then Homing (DH)	30	1 D then 1 H segment
Homing then Drifting (HD)	4	1 H then 1 D segment

While drifting only is the main diagnosed swimming behavior (n = 138), 30 turtles are diagnosed to switch from a drifting to a homing behavior (DH-trajectories) and 17 only display a homing behavior. Figure 9 shows that the automatic segmentation/classification algorithm does very well in detecting changes in the swimming behavior. It accurately identifies all long westwards homing segments that had been visually detected in Fig. 7 and places their beginning (breakpoint) at visually convincing positions. The mean duration of these H segments is close to one year (361 days) and the longest one is nearly 3 years long (1071 days). Their straightness is remarkable. It denotes a steady westward swimming activity that lasts until the track stops, consistent with the idea that, within these segments, turtles aim for a target west of their position.

More surprisingly, the automatic segmentation/classification algorithm also detects 4 trajectories in which turtles (all CNP turtles) switch from a homing to a drifting behavior (HD trajectories). These switches had not been visually detected but are now singled out in Fig. 9b. The interpretation of these trajectories will be discussed later (see Sect."Homing at what size?").

#### Meridional swimming activity

Large periodic movements, indicative of north–south seasonal migrations, are visible in Fig. 8 but their amplitudes and phases are largely blurred by estimation noise, especially in the case WNP turtles (Fig. 8b). The seasonal signal present in each trajectory can however be easily extracted from the background noise by fitting a sine curve of annual period to each meridional DC-trajectory. This is done using the SciPy curve_fit tool [84]. The amplitude and phase of the fitted sine curve are better estimated when the trajectory extends over, at least, one signal period (one year in this case). The NPLT data set includes 92 trajectories longer than a year. For these trajectories, the estimated phases (expressed as the date of the year when the sine curve reaches its maximum) have a circular mean corresponding to September 21. The mean amplitude of the fitted sine curves is 440 km, corresponding to a migration distance of 880 km (twice the amplitude). Migration distances below 500 km are observed in only 7 trajectories out of 92 and the smallest migration distance is 260 km. This simple analysis shows that juvenile NP loggerheads commonly perform very long-distance seasonal migrations phased in such a way that their northernmost positions are reached around the fall equinox.

**Fig. 10:**
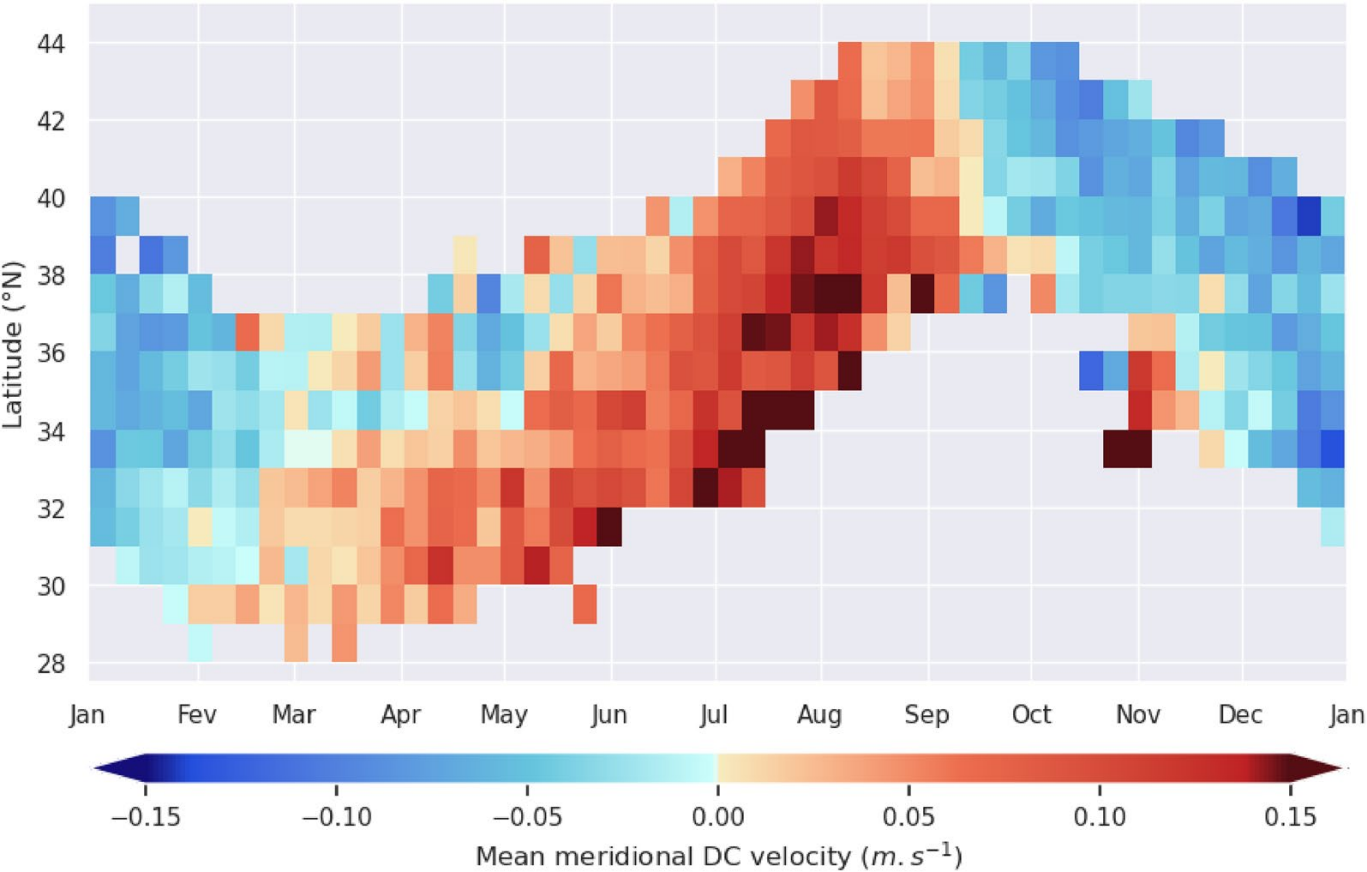
Mean meridional DC-velocities of loggerhead turtles as a function of date and latitude. Means are computed in [1° latitude × 7 days] boxes using all NPLT data. Only means based on more than 50 velocity data are shown

A more detailed discrete description of the seasonal evolution of meridional swimming velocities can be obtained sorting all meridional DC-velocity estimates in the NPLT dataset as a function of the date and latitude and then computing mean values in [1° latitude × 7 day] boxes (Fig. 10). The seasonal variability is clear: velocities are largely positive (northward) during spring and summer and negative during fall and winter. Their synchronicity is best marked in September when meridional velocities in nearly all latitude bands simultaneously change sign between week 37 and 38, confirming that the southward migration is initiated near the fall equinox and happens nearly synchronously, in individuals located at different latitudes. The springtime meridional velocity reversal is less clear-cut. It can appear as soon as February in individuals south of 30°N and later (until May) in individual north of 34 to 35°N.

#### Interactions between zonal and meridional swimming activities

**Fig. 11:**
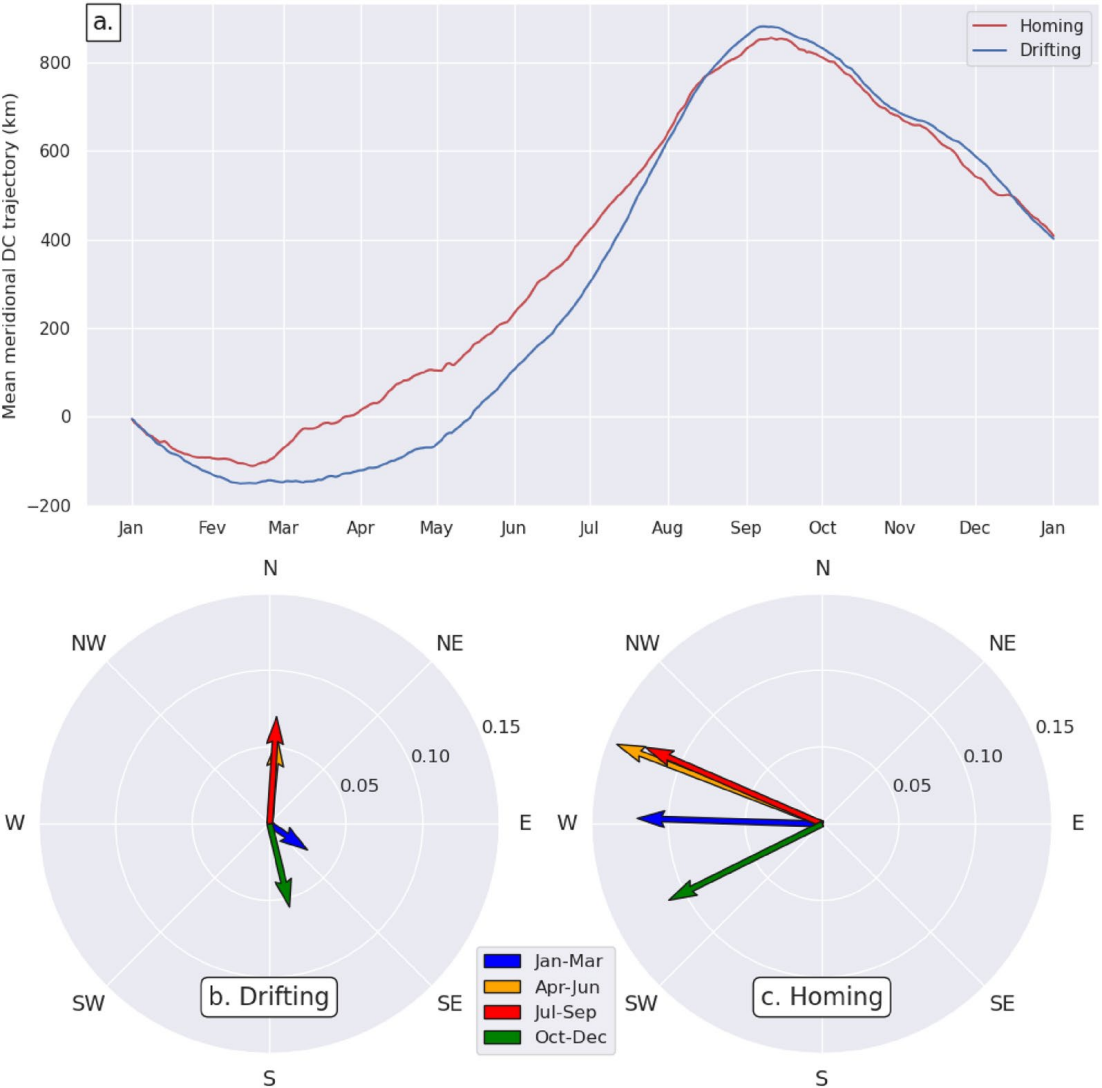
**a** Annual mean meridional DC-trajectories for drifting (blue) and homing (red) turtles; Seasonal mean DC-velocity vectors (m/s) for **b** drifting and **c** homing turtles

Both meridional migrations and zonal homing movements require considerable amounts of energy. One might thus expect homing juveniles to reduce the energy expended in meridional movements, that is to reduce the amplitude of their seasonal migrations when they need to invest more energy in zonal movements. To check this assertion, we sorted out as a function of date the meridional DC-velocities in both the D and H data set. A mean velocity was then computed for each day of the year. Finally, these mean meridional DC-velocities were numerically integrated with time, from January 1 st to December 31, to produce the annual mean meridional DC-trajectory of either drifting or homing turtles. Contrary to our expectations, these 2 DC-trajectories display seasonal cycles of comparable magnitude, with meridional migration distances of 967 km for homing turtles and 1032 km for drifting turtles (Fig. 11a). As a consequence, the total (zonal + meridional) swimming speed, and thus the turtles’ energy expense, appears to be systematically larger when homing than when drifting. This is clearly visible in Fig. 11b, c which show that the seasonal mean DC-velocity vectors computed using the H data set are, at least, twice as large as those derived from the D data set.

### The circumstances of homing

#### Is homing triggered seasonally?

**Fig. 12:**
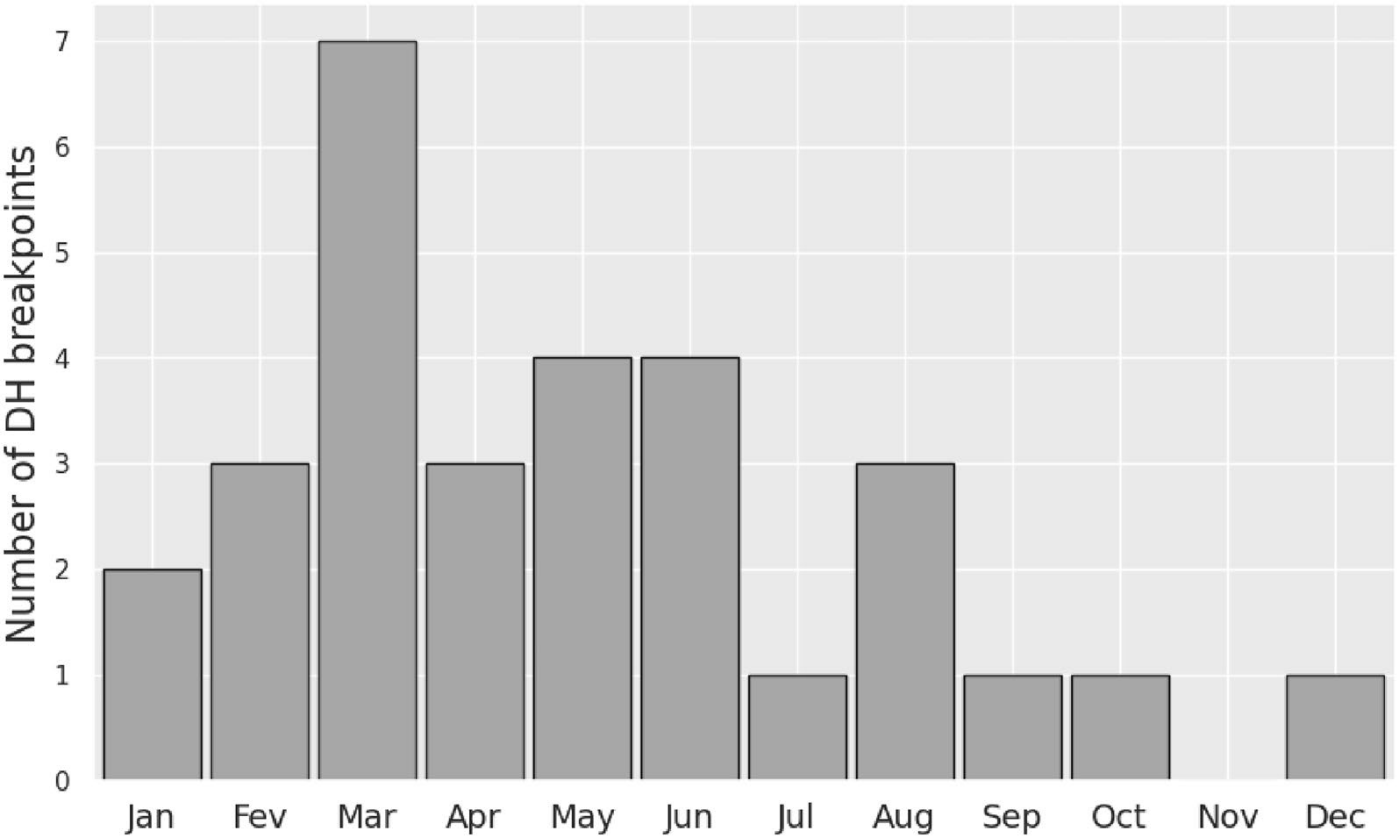
Annual distribution of the 30 DH breakpoints

The transition from a drifting to a homing behavior (DH breakpoint) is observed in 30 trajectories (Table 1). These events are more frequent during spring and rare during fall (Fig. 12). The hypothesis that their distribution is uniform over the year can clearly be rejected (Rayleigh test, p < 0.01). One reason for initiating homing during spring might be that, during that season, turtles are close to their southmost position, typically near 30°N, a latitude band where currents and Stokes drift are eastward but weaker than around 40°N (Fig. 4), the area where turtles are found during fall. It would then make sense to initiate westward movements when opposing currents are the weakest and avoid doing so when they are the strongest. More observations and further work are clearly needed to confirm or refute this hypothesis.

#### Homing at what size?

The precise mechanisms triggering the homing behavior in juvenile sea turtles are largely unknown but, like in other migrant species, evolution likely selected, and genetically encoded, one or several mechanisms that trigger that behavior at a time individuals have reached the developmental stage needed to undertake a long, energy consuming, homing journey [85]. Size being a good indicator of development, one expects homing to be triggered within a limited range of sizes corresponding to this developmental stage. This is indeed the case: 90%, that is 27 out of the 30 DH breakpoints, occur in juveniles with an SCL between 35 and 45 cm while only 53% of the NPLT data set falls within this size class. More precisely, the probability that homing occurred before a specified SCL, denoted ${F}_{H}\left(SCL\right),$ can be estimated using the complete set of valid segments, instead of breakpoints only. To achieve this, let us first define:

- [${S}_{min},{S}_{max}$] the range of SCL values for which ${F}_{H}\left(SCL\right)$ will be estimated,
- [${S}_{0},{S}_{1}$] the range of SCL values within which an individual is observed during a valid single segment or complete valid trajectory,
- ${S}_{b}$, the size (${S}_{0}<{S}_{b}<{S}_{1}$) at which a DH breakpoint (if any) occurs in a trajectory,
- $B$ the “behavioral state” (or swimming activity); it can be either “drifting”, “homing” or “unknown”.

Let us then assume that transition from a drifting to a homing behavior occurs only once during the pelagic juvenile phase. The 4 detected occurrences of HD breakpoints suggest that this single transition hypothesis is not always verified, but since HD breakpoints are infrequent compared to DH ones, it can be accepted to produce a first, probably gross, estimate of${F}_{H}\left(SCL\right)$. The 4 HD-trajectories being excluded $, B$ can be inferred as follows using all valid trajectories or trajectory segments displaying a unique D (n = 138) or H (n = 17) behavior and all DH-trajectories (n = 30):

i. For an individual only tracked drifting: $B$ is drifting in [${S}_{min},{S}_{1}$] and unknown in [${S}_{1},{S}_{max}$]
ii. For an individual only tracked homing: $B$ is unknown in [${S}_{min},{S}_{0}$] and homing in [${S}_{0},{S}_{max}$]
iii. For an individual tracked drifting than homing (DH trajectories): $B$ is drifting in [${S}_{min},{S}_{b}$] and homing in [${S}_{b},{S}_{max}$].

Then for any SCL value between ${S}_{min}$ and${S}_{max}$, all valid segments/trajectories can be inspected to determine ${n}_{H}$ and ${n}_{D}$, the number of them in which a homing or a drifting behavior is diagnosed for that SCL value. The estimated probability that homing occurred before SCL is then:

16 $${F}_{H}\left(SCL\right)={n}_{H}/({n}_{H}+{n}_{D})$$

**Fig. 13:**
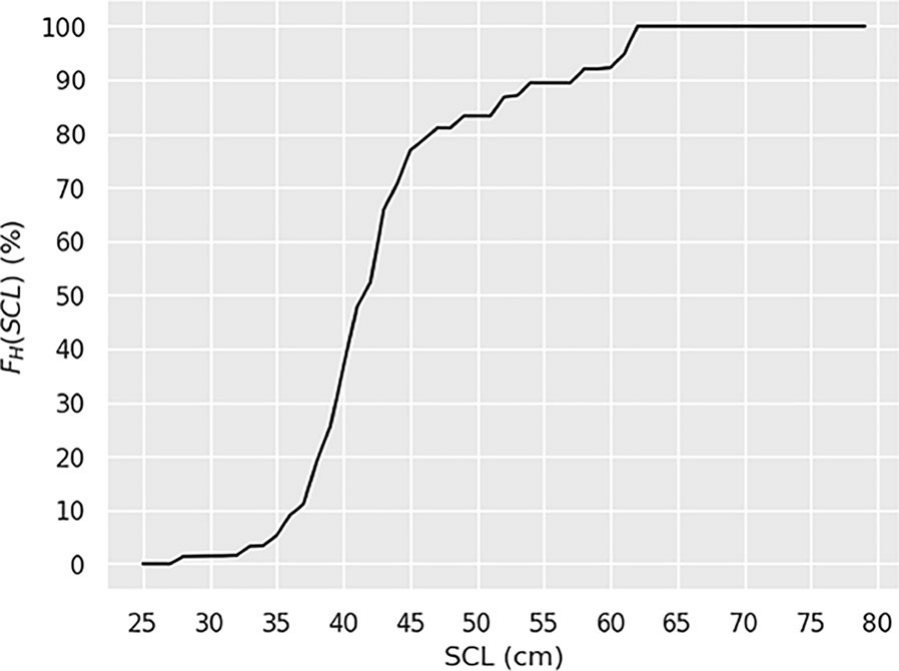
Estimated probability that homing occurred before a given SCL: ${F}_{H}\left(SCL\right)$. This probability is estimated for SCLs between ${S}_{min}$ = 25 cm and ${S}_{max}$ = 85 cm, with an SCL step of 1 cm

The resulting estimate is shown in Fig. 13. It confirms that the homing behavior is very unusual at SCLs < 35 cm. It indicates that about 50% the juveniles initiate homing before reaching the size of 42 cm and 80% of them before reaching an SCL of 50 cm. Above that size, the estimation accuracy is likely limited by the relatively small number of valid trajectories obtained for large individuals.

We should finally remember that Fig. 13 was obtained using all valid trajectories and segments of the NPLT data set, except for the 4 trajectories in which transition from a homing to a drifting behavior was observed. Occurrence of such a behavior is obviously rare. It might happen when the body condition of individuals swimming against adverse currents deteriorates, leading them to interrupt homing to adopt a less energy-consuming swimming behavior, possibly before later resuming their westward journey. Similar events of aborted migrations are observed in birds encountering adverse environmental conditions [86, 87]. It might be the type of event we observe here. Indeed, the 4 turtles displaying a HD-trajectory were all small at the time of release (32.3 cm < SCL < 34.5 cm), a size range within which homing is unusual (Fig. 13). However, they were probably in excellent body condition as they had just left the aquarium where they had been captive-reared. Their high energy stores might thus have prompted them to initiate homing early in their development cycle. Released in the wild and immediately swimming westwards against the currents, their body condition could deteriorate, finally leading them to adopt a less active behavior, better adapted to their size and energy intake at sea. Note however that their “less active” behavior still involves well-marked meridional migrations over distances around 1000 km, maintained throughout their whole tracking period.

#### Homing trajectories: a subtle balance between westward swimming and eastward drift

**Fig. 14:**
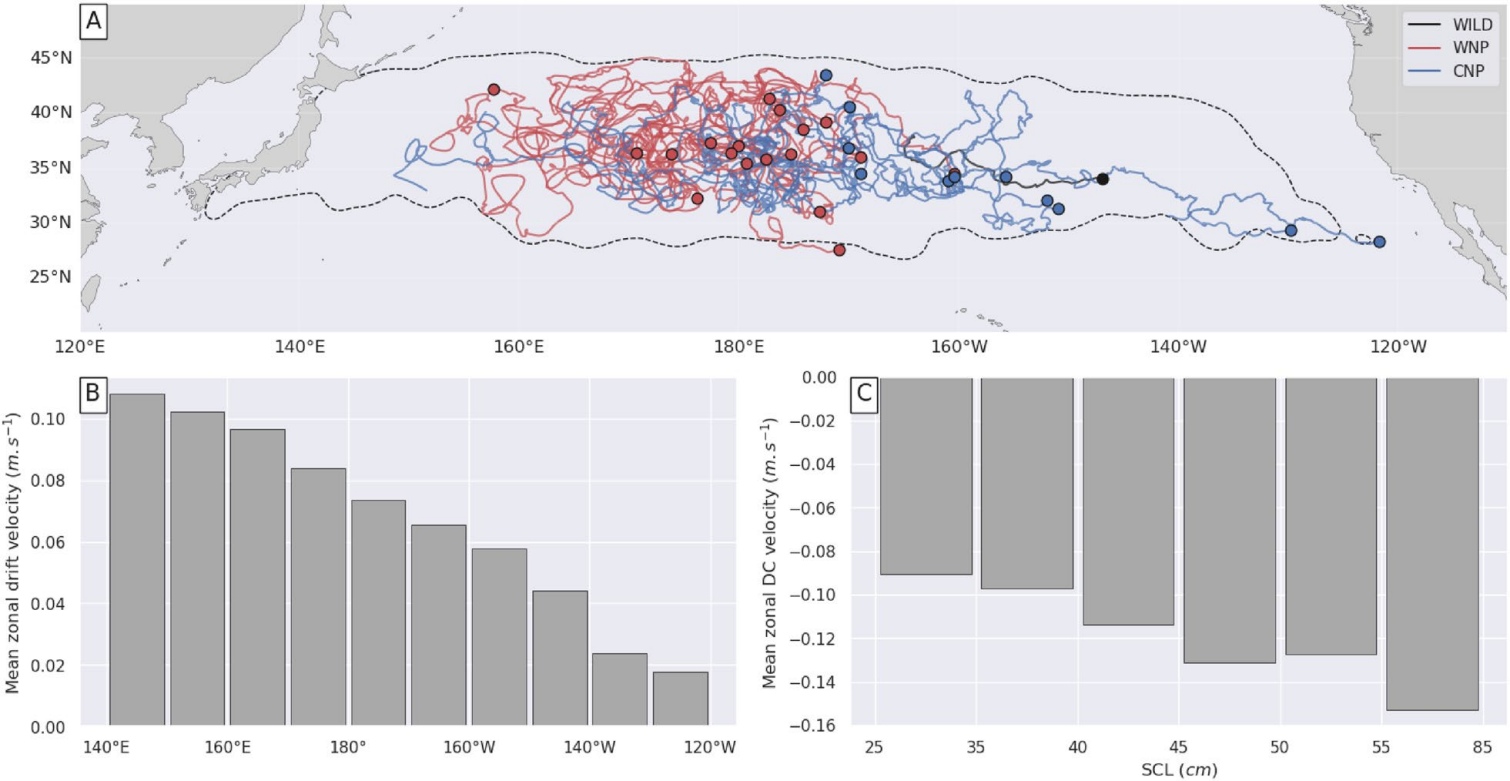
**a** Map of the homing segments of the 30 DH trajectories with their initial breakpoint positions (small circles), in red for WNP turtles, blue for CNP turtles and black for wild turtles. The dotted black line delineates the area with the highest density of recorded turtle positions (97% of all turtle positions in the NPLT data set are found inside this area); **b** Mean zonal drift velocities as a function of longitude computed with Eq. (14b) using modeled surface currents and Stokes drift data averaged in 10° longitude boxes between 30 and 45°N; **c** Mean zonal DC-velocities as a function of size for homing turtles, based on all zonal DC-velocities in the H dataset

In the latitude range (~ 30 to 45°N) where homing occurs (Fig. 14a) ocean circulation is clearly eastward (Fig. 4). Mean zonal drift velocities are relatively small (around 4 cm/s) in the Eastern Pacific but steadily increase going west, reaching 8 cm/s near the dateline, and over 11 cm/s near the Japanese coastline (Fig. 14b). Homing turtles swim in the opposite direction with westwards velocities typically between −9 and −13 cm/s, becoming more negative with size. They reach −15 cm/s only in the few tracked individuals with SCL > 55 cm (Fig. 14c). With such swimming speeds, homing individuals of all sizes can easily progress against the weak adverse currents found in the easternmost part of the tracking area. Their trajectories are rather straight and clearly westwards (Fig. 14a). Further west, eastward currents become stronger, reaching speeds comparable, but opposite in sign, to those of homing turtles. As a result, turtles’ zonal velocities over ground become small, often oscillating between positive and negative values. This generates the very convoluted homing trajectories observed in the Western Pacific (Fig. 14a), displaying both eastward and westward movements.

**Fig.15:**
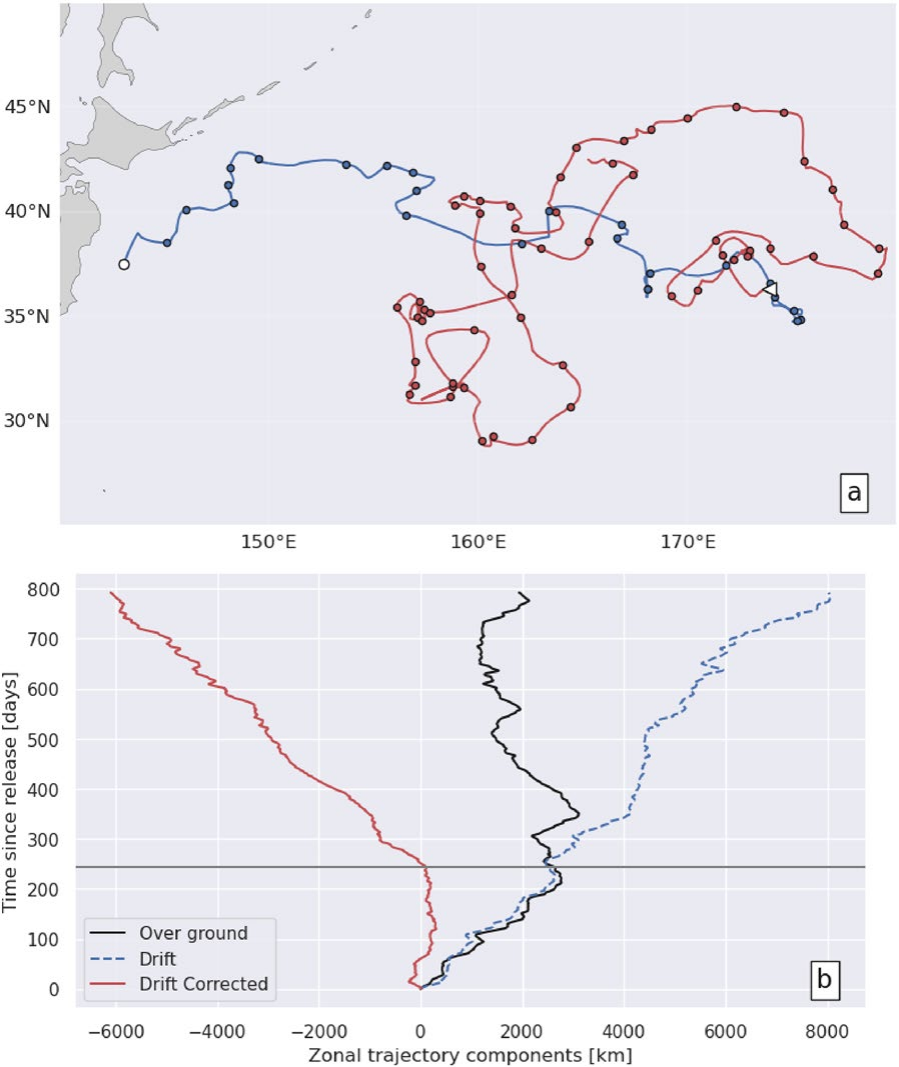
**a** D (blue) and H (red) segments of the trajectory over ground of a turtle released off Japan in July 2010. The small white triangle shows the position of the breakpoint detected in this trajectory. Small dots along the trajectories are turtle positions every 10 days. **b** Zonal components of this turtle’s trajectory over ground (solid black line), drift trajectory (${\int }_{{t}_{0}}^{t}{u}_{d}dt$, blue dotted line), and DC-trajectory (red solid line). The horizontal black line at t = 244 days corresponds to the date of the breakpoint

Such trajectories are naturally associated with long residence times in the WNP. A typical example is provided with the trajectory of a medium-sized juvenile (SCL = 39.1 cm) released off Japan in July 2010 (Fig. 15). This turtle was first observed drifting for 244 days from 143°E to 174°E, the longitude at which it initiated homing at an estimated SCL = 41,7 cm. It was then tracked for another 549 days until its tag stopped working at 165.3°E, less than 10° west of the position where it started homing. The homing segment of its trajectory over ground (Fig. 15a) is very tortuous with periods during which the turtle alternately gains or loses ground against opposing currents. Large seasonal north–south movements are also visible on top of smaller-scale oscillations and loops typically induced by oceanic mesoscale features. On the contrary, its zonal DC-trajectory (Fig. 15b) is rather straight throughout the whole homing phase, revealing that the turtle constantly maintains a fairly stable westward swimming activity despite the large variability of the encountered currents. As in this example, most turtles diagnosed to be homing manage to make headway despite opposite currents and are closer to Japan at the end of their homing segment than at the beginning, but this is not always the case. The worst case is that of a WNP turtle which lost (i.e. moved eastward) over 20° in longitude during its observed 2-year-long homing segment. This turtle had the westernmost diagnosed breakpoint (157.7°E) in the whole NPLT data set and initiated homing at a rather small size (SCL = 36 cm). Being small (and thus a weaker swimmer) and west of the dateline are two conditions that clearly make the homing journey most difficult.

## Discussion

### Drifting then homing seasonal migrations (DHSM)

Analysis of DC-trajectories reveals that juvenile NP loggerhead turtles display two types of large-scale, long-term swimming movements: seasonal meridional migrations and westward homing movements. Seasonal movements are present in individuals of all sizes while homing movements gradually appear as individuals grow. Before their onset, juveniles have no significant zonal swimming activity and appear to drift zonally with the prevailing eastward currents while undertaking large north–south seasonal migrations. This period of drifting seasonal migrations, ends when juveniles initiate homing. They then perform homing seasonal migrations as they maintain large meridional seasonal migrations but with an added westward swimming component.

This sequence of drifting then homing seasonal migrations (DHSM) constitutes the basic swimming scenario that governs juvenile loggerheads dispersal in the North Pacific. It likely unfolds over time as follows. Hatchlings leaving their natal beach in Japan are first entrained north-eastwards, along the Japanese coast, by the Kuroshio current. Within a few months, they progressively veer east, following the Kuroshio that detaches from the coast around 35°N [88], and initiate their crossing of the North Pacific basin. If drifting purely passively, they could reach latitudes between 40 and 45°N during their first winter at sea [12] where they might suffer cold-induced mortality if they encounter water temperatures well below 10 °C [89]. The mortality rate should however be relatively low. Indeed, sea surface temperatures (SST) from the GLORYS12 reanalysis [63], extracted along the trajectories of the surface buoys that passed off Japan during the hatching season (mid-July to mid-October), show that only 16% of these buoys encounter sea surface temperatures below 10 °C during the following winter. If small juveniles already swim southwards during their first fall at sea, the risk should even be smaller.

During subsequent years, juveniles will progress across the NP basin, drifting eastwards with the Kuroshio extension current and then the North Pacific current. Cold-stunning after the first winter at sea is less likely as seasonal migrations lead individuals southwards, well inside the gyre, from the beginning of the fall till the end of the winter. After several years of eastward drift and seasonal migrations, juveniles will finally approach Northern California and enter the California Current System (CCS). It will entrain them southwards towards Baja California Peninsula (BCP) and its rich neritic foraging grounds where over 40,000 juvenile or sub-adult loggerheads are observed to forage all year round [90]. Juvenile loggerheads having reached BCP are observed to reside there for several years, switching from a pelagic to a neritic foraging strategy [91].

Reaching the BCP foraging grounds is not guaranteed, however. Recent work suggest that crossing of the cold California Current System might only be possible through an occasional corridor of anomalously warm waters [45]. In addition, during their crossing of the NP, many juveniles might become large enough to initiate homing. They would thus return towards Japan before reaching California. The smallest individuals reaching BCP have SCLs close to 30 cm but most individuals arrive at sizes between 40 and 65 cm SCL [91, 92]. Within this range of sizes, our results suggest that 40 to 100% of the juveniles have already initiated their homing journey (Fig. 13). This means that homing could be initiated by individuals close to reaching BCP but it might also occur at more westerly positions in individuals that drifted more slowly or grew faster.

Individuals initiating homing before reaching BCP are expected to return towards Japan following trajectories similar to those observed in the NPLT data set, that is swimming westwards against the dominant currents and migrating seasonally. This will be relatively easy for the largest, most powerful, individuals but more difficult for the smaller ones.

The story is a bit different for juveniles reaching BCP as their residence time in this area can reach up to 20 or more years [91]. This represents a different, but well-known, development strategy in which individuals take advantage of remote neritic feeding areas before moving back to their natal area. These individuals will thus be larger and more powerful swimmers when initiating their homeward journey. A fraction of the large wild turtles of the NPLT data set likely are individuals that resided in BCP before being captured, tagged, and released on their way back to Japan. Their westwards swimming velocities are among the fastest (~ 15 cm/s) observed in the NPLT data set but their trajectories are similar to those of homing WNP or CNP individuals suggesting that the same way back is used by all homing juveniles whether having reached BCP or not. This way back is against the dominant eastwards current. None of the observed individuals seeks to reach the Pacific North Equatorial current that flows westwards at much lower latitudes. Interestingly, a larger sub-adult directly tracked from BCP displayed the same behavior, returning directly to Japan [93] against the current.

Alongside this description of the likely dispersal scenario of juvenile NP loggerheads, two points are worth noting:

i. Fig. 3b shows that the minimum time needed for WNP buoys to cross the whole Pacific Ocean is about 2.5 to 3 years while the smallest individuals reaching BCP have an SCL close to 30 cm, thus an age close to 4.5 years according to the Ramirez et al. [77] growth curve, using ${L}_{o}$ = 4.2 cm, the mean size of hatchlings in the NP loggerhead population [94]. This is additional evidence that passive drift times are generally too short to explain the time taken by juvenile sea turtles to cross oceanic basins.
ii. Most juveniles reaching BCP have an SCL > 40 cm [91, 92], a size at which our results suggest that about 40% of the individuals have already initiated homing and will thus never make it to BCP. The percentage of individuals initiating homing before reaching BCP should thus be much larger among our tracked reared individuals. WNP turtles, released off Japan with an SCL > 25 cm, have virtually no chance of reaching BCP before initiating homing. CNP turtles released at sizes close to that of the smallest wild individuals reaching BCP, are also less likely than wild turtles to reach BCP. This certainly contributes explaining why, in the NPLT data set, only 2 of the 198 captive-reared turtles reach the BCP area, and these are CNP turtles.

### Supporting the cost of swimming

The DHSM dispersal scenario is a very active one. Achieving a 900-km migration over a 6-month period requires a mean meridional swimming velocity close to 6 cm/s. The swimming velocity of homing individuals includes an additional westward component which ranges from 9 cm/s in the smallest individuals up to 15 cm/s in the largest ones (Fig. 14c). The total swimming velocity of homing individuals is thus easily twice as large as the velocity of drifting individuals, as already observed in Fig. 11 b,c. As the energy expended per second to maintain a velocity is proportional to the cube of that velocity (e.g. [95]), doubling the velocity multiplies by 8 the rate of energy expended for moving. Juveniles thus have to support much larger energy expenditures when homing than when drifting.

In reality, large, fast swimming, individuals will be found in the western Pacific, the largest/oldest ones finally reaching the Japanese waters. The most difficult part of their westward progression takes place west of the date line where adverse eastward currents are the strongest (Fig. 14b). Individuals tracked in this area are generally able to make headway against these currents, albeit very slowly and along very tortuous trajectories (Figs. 14a, 15). The western Pacific, and the KEBR area in particular, is a highly energetic but also very productive area [40]. Its high productivity most probably is a critical factor making homing possible as abundant refuelling opportunities are made available in a zone where individuals must maintain high velocities and thus sustain high energy costs. Foraging activities and long residence times detected in the KEBR [40, 43] must be placed in that context. They should not be interpreted as a sign that juvenile loggerheads simply are long term residents, foraging in this rich area, but rather transiting individuals that struggle to cross the KEBR against strong adverse currents while foraging to support the cost of their high swimming activity.

### Underlying navigation mechanisms

Large-scale seasonal migrations are widespread across taxonomical groups and different navigational toolkits likely guiding them have been identified [96]. However, the drifting and then the homing seasonal migrations of the juvenile NP loggerhead turtles, both maintained over several years, are unusual. The navigational tools and strategies enabling such migrations still have to be precisely identified. This is far beyond the scope of this paper but some of our results provide preliminary clues.

During their initial drifting phase, individuals swim alternately north and south depending on the season (Fig. 11b). This behavior could be enabled by the use of one of the simplest and most widespread orientation technique among all taxa, the so-called clock-and-compass method [96, 97]. It relies on an inherited migratory direction that individuals follow using a celestial or magnetic compass during an innate period of time. This period is controlled by a circannual clock triggering the start/stop of the migration legs [98, 99]. To make use of that technique, juvenile loggerheads must possess an innate migration direction, a compass and an endogenous circannual clock. Assuming that NP loggerheads inherit a single (North–South) migration direction seems reasonable as North Atlantic loggerheads are known to inherit an even more complex set of location-dependent swimming directions [28]. Like birds [100], loggerheads are also known to possess an inclination compass [101] which is well adapted to guide their North–South migrations.

In the original formulation of the clock-and-compass mechanism, the circannual clock is endogenous but avian migration studies have shown that exogenous time-keeping cues also often play a major role in migration timing [102]. Whether or not sea turtles possess an endogenous circannual clock can be debated but, in any case, turtles have access to external cues allowing them to remain synchronized with seasons. The most obvious one is the day length which is known to be used, at least for circadian time keeping, in green turtles [103]. In temperate areas, water temperature also constitutes a clear seasonal indicator. These cues could certainly be used to trigger reversals of the migration direction. As discussed earlier, our results show that the fall reversal of the migration direction is highly synchronous in juvenile NP loggerheads: it happens between week 37 and 38 (that is a few days from the equinox) in most individuals, then located at latitudes between 37 and 44°N (Fig. 10). Such a strict timing might be the result of a strong evolutionary pressure pushing individuals to start swimming southwards at the beginning of the fall before water temperatures start decreasing too rapidly. The fact that the fall migration is initiated almost simultaneously in individuals located several degrees of latitude apart suggest that the cue triggering the reversal of the migration direction is little or not dependent on the latitude. This is the case for day length at the time of the equinox, but not for water temperature or Earth magnetic field values. The day length is thus a most likely cue triggering NP loggerheads’ southward fall migration.

The onset of the spring migration does not seem to be under the same pressure as mean meridional velocity reversals are observed to occur over a longer period, mostly between mid-February and the mid-May. Instead of initiating their northwards spring migration at (or near) a fixed date, turtles could benefit from following a more flexible schedule governed by environmental cues, food abundance in particular. A number of studies have indeed shown that NP juvenile loggerheads appear to track the TZCF, a basin-wide chlorophyll front that serves as a proxy for the concentration of their prey [41, 80, 81]. This front represents the boundary between the low chlorophyll subtropical gyre and the high chlorophyll subarctic gyre. It moves seasonally from the south of the NP Transition Zone in the winter to the north of it in the summer [81]. Habitat models that use a proxy for the TZCF together with sea surface temperature largely capture the seasonal meridional movement of loggerheads [41, 42]. It might thus very well be that NP loggerhead seasonal migrations are shaped by a combination of habitat-driven movements and movements driven by a compass and a clock largely relying on the photoperiod. Habitat-driven movements might dominate during spring and summer when feeding is a priority while compass-driven movements should become more important during fall/winter when southward movements are mandatory to ensure critical water temperatures are avoided. A similar navigation scheme was previously suggested by Kobayashi et al. [41], who argued that a magnetic compass could serve as the general guide for large scale migrations while local environmental cues would be used to constrain small-scale movements allowing appropriate use of local resources.

Homing requires different, probably more elaborate, navigations strategies than those used for seasonal migrations. Homing can typically be achieved using either route-based or map-based navigation [104]. Route-based navigation is achieved on the basis of information perceived during the outward journey from the known goal to the location where homing is initiated. This type of navigation can be excluded here as none of the homing CNP turtles previously performed the outward journey. Map-based navigation is much more likely. It requires turtles to assess the direction of their natal area relative to their position and then head in that direction (grossly west in this case). They do so in an unexpected and rather complex manner as, for probably several years, they maintain a steady westward component in their swimming velocity vector while still performing large-scale meridional migrations. To the best of our knowledge, similar pluriannual “oscillating” homing migrations have not been observed in other animals. The navigation strategy used to achieve this fascinating homeward journey remains to be figured out but probably still relies on a magnetic compass and a clock that keeps the same migration timing as the one used during drifting migrations.

## Conclusion

A novel method using sea turtle and passive drifter tracking data together with operational ocean model outputs has been proposed here to reliably separate, at least on the large scale, the turtles’ swimming velocity from the drift velocity, the latest being generated not only by oceanic currents but also by the windage and Stokes drift.

This method has been applied to the exceptionally dense NPLT data set to identify and study the swimming activity of juvenile NP loggerheads during most of their oceanic phase. Results reveal that the smallest tracked individuals have no significant large-scale zonal swimming activity. They mostly drift zonally with the prevailing eastward currents while undertaking large (~ 1000 km) north–south seasonal migrations. These migrations take them well inside the NP subtropical gyre during each fall and winter, thereby largely avoiding the risk of cold-stunning and the risk of straying out of the gyre. This period of drifting seasonal migrations, ends when juveniles initiate homing, most of them at sizes between 35 and 45 cm SCL. They then start swimming westwards towards Japanese waters against the prevailing eastward currents. They do so while maintaining large meridional seasonal migrations. This very active return journey progressively requires more and more energy as westward-moving individuals become bigger and faster swimmers, progressively encountering stronger eastward currents when they re-enter the western Pacific basin. This very dynamic ocean basin, is richer than the Eastern Pacific, thereby opportunely providing better foraging opportunities.

It thus appears that rather than minimizing their energy expenditure by adopting a mostly passive migration strategy, juvenile NP loggerheads have evolved the very active DHSM swimming strategy that shapes their spatial dispersal in such a way that high energy expenditure can timely be offset by matching energy intake. The initial zonal drifting phase is the least active part of this swimming scenario during which seasonal migrations towards northern summer foraging grounds likely ensure reliable food intake, at least on an annual basis. The more active homing phase is facilitated by increased productivity in the western NP which provides more abundant foraging opportunities in areas where energy needs are greatest.

The NPLT data set, and the DHSM swimming scenario it reveals, cover a large fraction of the juvenile NP loggerhead oceanic dispersal phase, but not all of it. The NPLT data set is too scarce east of 130°W to allow a detailed understanding of how many juvenile loggerheads reach Baja California. This is the objective of the ongoing STRETCH project (https://www.loggerheadstretch.org/) which will provide unique information complementing the data analysed here. NPLT data are also lacking to adequately cover the very first months at sea of juvenile loggerheads. Tracking of very small loggerheads is now possible [9, 34] and, when achieved, will tell us (among others) if juveniles have already initiated seasonal migrations at the time of their first winter at sea.

Finally, given the variability of the physiological and environmental constraints acting on different sea turtle species and populations in different ocean basins, we should expect that sea turtles have evolved a wide range of juvenile swimming scenarios shaping their oceanic dispersal phase. The main cues triggering or enabling the different phases of these scenarios should also vary between the different populations and ocean basins. For example, the day length likely is less informative and the water temperature less critical in equatorial areas than at mid-latitudes. In any case, our novel approach to separate the turtles’ swimming velocity from the drift velocity should prove useful to uncover more of these swimming scenarios. To achieve that goal, new long-term tracking experiments involving juvenile sea turtles are critically needed.

## Supplementary Information

Additional file 1.

## Data Availability

This study has been conducted using publicly available E.U. Copernicus Marine Service Information, in particular GLORYS12 surface currents (https://doi.org/10.48670/moi-00021), WAVERYS surface Stokes drift data (https://doi.org/10.48670/moi-00022) and processed Global Drifter Program Data (https://doi.org/10.17882/86236). The juvenile loggerhead tracking data used here are available from Denise Parker (denise.m.parker@outlook.com) on reasonable request.

## Abbreviations

BCP: Baja California Peninsula
CNP: Central North Pacific
DC: Drift-corrected (velocities, trajectories)
D, H: Drifting or homing (behavior, trajectories or trajectory segments)
DHSM: Drifting then homing seasonal migrations
KEBR: Kuroshio extension bifurcation region
NP: North Pacific
NPLT: North Pacific loggerhead tracking (data set)
SCL: Straight carapace length
TZCF: Transition zone chlorophyll front
WNP: Western North Pacific

## References

[1] Musick JA, Limpus CJ. Habitat utilization and migration in juvenile sea turtles. The biology of sea turtles. Boca Raton: CRC Press; 1997. p. 137–64.

[2] Carr A. Rips, FADS, and little loggerheads. Bioscience. 1986;36(2):92–100.

[3] Bolten AB, Bjorndal KA, Martins HR, Dellinger T, Biscoito MJ, Encalada SE, et al. Transatlantic developmental migrations of loggerhead sea turtles demonstrated by mtDNA sequence analysis. Ecol Appl. 1998;8(1):7.

[4] Bowen BW, Abreu-Grobois FA, Balazs GH, Kamezaki N, Limpus CJ, Ferl RJ. Trans-Pacific migrations of the loggerhead turtle (Caretta caretta) demonstrated with mitochondrial DNA markers. Proc Natl Acad Sci. 1995;92(9):3731–4. 7731974 10.1073/pnas.92.9.3731 PMC42035

[5] Boyle MC, FitzSimmons NN, Limpus CJ, Kelez S, Velez-Zuazo X, Waycott M. Evidence for transoceanic migrations by loggerhead sea turtles in the southern Pacific Ocean. Proceed Royal Soc B: Biol Sci. 2009;276:1993–9. 10.1098/rspb.2008.1931 PMC2677240 19324768

[6] Carreras C, Pont S, Maffucci F, Pascual M, Barceló A, Bentivegna F, et al. Genetic structuring of immature loggerhead sea turtles (Caretta caretta) in the Mediterranean Sea reflects water circulation patterns. Mar Biol. 2006;149(5):1269–79.

[7] Monzón-Argüello C, López-Jurado LF, Rico C, Marco A, López P, Hays GC, et al. Evidence from genetic and Lagrangian drifter data for transatlantic transport of small juvenile green turtles: Green turtle dispersal in the Atlantic. J Biogeogr. 2010;37(9):1752–66.

[8] Polovina JJ, Kobayashi DR, Parker DM, Seki MP, Balazs GH. Turtles on the edge: movement of loggerhead turtles (Caretta caretta) along oceanic fronts, spanning longline fishing grounds in the central North Pacific, 1997–1998. Fish Oceanogr. 2000;9(1):71–82.

[9] Mansfield KL, Wyneken J, Porter WP, Luo J. First satellite tracks of neonate sea turtles redefine the “lost years” oceanic niche. Proceed Royal Soc B: Biol Sci. 2014;281(1781):20133039–20133039. 10.1098/rspb.2013.3039 PMC3953841 24598420

[10] Putman NF, Mansfield KL. Direct evidence of swimming demonstrates active dispersal in the sea turtle “lost years.” Curr Biol. 2015;25(9):1221–7. 25866396 10.1016/j.cub.2015.03.014

[11] Briscoe DK, Parker DM, Balazs GH, Kurita M, Saito T, Okamoto H, et al. (2016) Active dispersal in loggerhead sea turtles (*Caretta caretta* ) during the ‘lost years. Proceedings of the Royal Society B Biological Sciences. 283(1832): 20160690. 10.1098/rspb.2016.0690 PMC4920322 27252021

[12] Okuyama J, Kitagawa T, Zenimoto K, Kimura S, Arai N, Sasai Y, et al. Trans-Pacific dispersal of loggerhead turtle hatchlings inferred from numerical simulation modeling. Mar Biol. 2011;158(9):2055–63.

[13] Gaspar P, Benson S, Dutton P, Réveillère A, Jacob G, Meetoo C, et al. Oceanic dispersal of juvenile leatherback turtles: going beyond passive drift modeling. Mar Ecol Prog Ser. 2012;21(457):265–84.

[14] Putman NF, Mansfield KL, He R, Shaver DJ, Verley P. Predicting the distribution of oceanic-stage Kemp’s ridley sea turtles. Biol Let. 2013;9(5):20130345–20130345. 23945206 10.1098/rsbl.2013.0345 PMC3971672

[15] Putman NF, Naro-Maciel E. Finding the “lost years” in green turtles: insights from ocean circulation models and genetic analysis. Proceed Royal Soc B: Biol Sci. 2013;280(1768):20131468–20131468. 10.1098/rspb.2013.1468 PMC3757977 23945687

[16] Scott R, Marsh R, Hays G. Ontogeny of long distance migration. Ecology. 2014;25:140425082009005.

[17] Vandeperre F, Parra H, Pham CK, Machete M, Santos M, Bjorndal KA, et al. Relative abundance of oceanic juvenile loggerhead sea turtles in relation to nest production at source rookeries: implications for recruitment dynamics. Sci Rep. 2019;9(1):13019. 31506566 10.1038/s41598-019-49434-0 PMC6737082

[18] Scott R, Marsh R, Hays GC. Life in the really slow lane: loggerhead sea turtles mature late relative to other reptiles: *Reptile age at maturity*. Funct Ecol. 2012;26(1):227–35.

[19] Maximenko N, Hafner J, Niiler P. Pathways of marine debris derived from trajectories of Lagrangian drifters. Mar Pollut Bull. 2012;65(1–3):51–62. 21696778 10.1016/j.marpolbul.2011.04.016

[20] Mansfield KL, Putman NF. 2013 Oceanic Habits and habitats Caretta caretta In The biology of sea turtles, vol 3. CRC Press/Taylor & Francis Group. p. 189–210.

[21] Lohmann KJ. Regional magnetic fields as navigational markers for sea turtles. Science. 2001;294(5541):364–6. 11598298 10.1126/science.1064557

[22] Putman NF, Verley P, Shay TJ, Lohmann KJ. Simulating transoceanic migrations of young loggerhead sea turtles: merging magnetic navigation behavior with an ocean circulation model. J Exp Biol. 2012;215(11):1863–70. 22573765 10.1242/jeb.067587

[23] Varo-Cruz N, Bermejo JA, Calabuig P, Cejudo D, Godley BJ, López-Jurado LF, et al. 2016 New findings about the spatial and temporal use of the Eastern Atlantic Ocean by large juvenile loggerhead turtles. Roura-Pascual N, editor. Diversity and Distributions 22(4): 481–92.

[24] Chambault P, Baudena A, Bjorndal KA, Santos MAR, Bolten AB, Vandeperre F. Swirling in the ocean: Immature loggerhead turtles seasonally target old anticyclonic eddies at the fringe of the North Atlantic gyre. Prog Oceanogr. 2019;175:345–58.

[25] Cejudo D, Varo-Cruz N, Liria A, Castillo JJ, Jesús J, López-Jurado LF. Transatlantic migration of juvenile loggerhead turtles (*Caretta caretta* L.) from the strait of Gibraltar. Mar Turt Newsl. 2006;114:9–11.

[26] Lohmann KJ, Witherington B, Lohmann CMF, Salmon M. Orientation, navigation and natal beach homing in sea turtles. In: The biology of sea turtles. Boca Raton: CRC Press; 1997. p. 107–35.

[27] Fuxjager MJ, Eastwood BS, Lohmann KJ. Orientation of hatchling loggerhead sea turtles to regional magnetic fields along a transoceanic migratory pathway. J Exp Biol. 2011;214(15):2504–8. 21753042 10.1242/jeb.055921

[28] Lohmann KJ, Putman NF, Lohmann CM. The magnetic map of hatchling loggerhead sea turtles. Curr Opin Neurobiol. 2012;22(2):336–42. 22137566 10.1016/j.conb.2011.11.005

[29] Putman NF, Endres CS, Lohmann CMF, Lohmann KJ. Longitude perception and bicoordinate magnetic maps in sea turtles. Curr Biol. 2011;21(6):463–6. 21353561 10.1016/j.cub.2011.01.057

[30] Putman NF, Verley P, Endres CS, Lohmann KJ. Magnetic navigation behavior and the oceanic ecology of young loggerhead sea turtles. J Exp Biol. 2015;218(7):1044–50. 25833134 10.1242/jeb.109975

[31] Gaspar P, Lalire M. A model for simulating the active dispersal of juvenile sea turtles with a case study on western Pacific leatherback turtles. Hays G, editor. PLoS ONE. 2017;12(7):e0181595. 28746389 10.1371/journal.pone.0181595 PMC5528265

[32] Lalire M, Gaspar P. Modeling the active dispersal of juvenile leatherback turtles in the North Atlantic Ocean. Mov Ecol. 2019;7(1):7. 30858978 10.1186/s40462-019-0149-5 PMC6394021

[33] Mansfield K, Wyneken J, Rittschof D, Walsh M, Lim C, Richards P. Satellite tag attachment methods for tracking neonate sea turtles. Mar Ecol Prog Ser. 2012;21(457):181–92.

[34] Candela T, Wyneken J, Leijen P, Gaspar P, Vandeperre F, Norton T, et al. Novel microsatellite tags hold promise for illuminating the lost years in four sea turtle species. Animals. 2024;14(6):903. 38540001 10.3390/ani14060903 PMC10967317

[35] Chambault P, Gaspar P, Dell’Amico F. Ecological trap or favorable habitat? First evidence that immature sea turtles may survive at their range-limits in the North-East Atlantic. Front Mar Sci. 2021;26(8): 736604.

[36] Barbour N, Bailey H, Fagan WF, Mustin W, Baboolal V, Casella F, et al. Satellite tracking of head-started juvenile green turtles (Chelonia mydas) reveals release effects and an ontogenetic shift. Animals. 2023;13(7):1218. 37048474 10.3390/ani13071218 PMC10093175

[37] Vidal A. Growing pains: investigating satellite tag epoxy attachments on juvenile turtles. University of West Florida; 2021 p. 87. (Master Thesis).

[38] Fossette S, Putman N, Lohmann K, Marsh R, Hays G. A biologist’s guide to assessing ocean currents: a review. Mar Ecol Prog Ser. 2012;21(457):285–301.

[39] Mansfield KL, Mendilaharsu ML, Putman NF, dei Marcovaldi MAG, Sacco AE, Lopez G, et al. 2017 First satellite tracks of South Atlantic sea turtle ‘lost years’: seasonal variation in trans-equatorial movement. Proc R Soc B. 1868;284:20171730. 10.1098/rspb.2017.1730 PMC5740273 29212722

[40] Polovina J, Uchida I, Balazs G, Howell EA, Parker D, Dutton P. The Kuroshio extension Bifurcation region: a pelagic hotspot for juvenile loggerhead sea turtles. Deep Sea Res Part II. 2006;53(3–4):326–39.

[41] Kobayashi DR, Polovina JJ, Parker DM, Kamezaki N, Cheng IJ, Uchida I, et al. Pelagic habitat characterization of loggerhead sea turtles, Caretta caretta, in the North Pacific Ocean (1997–2006): Insights from satellite tag tracking and remotely sensed data. J Exp Mar Biol Ecol. 2008;356(1–2):96–114.

[42] Abecassis M, Senina I, Lehodey P, Gaspar P, Parker D, Balazs G, et al. A Model of Loggerhead sea turtle (Caretta caretta) Habitat and movement in the oceanic North Pacific. Hays GC, editor. PLoS ONE. 2013;8(9):e73274. 24039901 10.1371/journal.pone.0073274 PMC3764129

[43] Briscoe DK, Parker DM, Bograd S, Hazen E, Scales K, Balazs GH, et al. Multi-year tracking reveals extensive pelagic phase of juvenile loggerhead sea turtles in the North Pacific. In: Movement ecology [Internet]. 2016 Dec [cited 2018 Dec 14];4(1). Available from: http://movementecologyjournal.biomedcentral.com/articles/10.1186/s40462-016-0087-4 10.1186/s40462-016-0087-4 PMC5048666 27729983

[44] Putman NF, Lumpkin R, Sacco AE, Mansfield KL. Passive drift or active swimming in marine organisms? Proceed Royal Soc B: Biol Sci. 2016;283(1844):20161689. 10.1098/rspb.2016.1689 PMC5204149 27974518

[45] Briscoe DK, Turner Tomaszewicz CN, Seminoff JA, Parker DM, Balazs GH, Polovina JJ, et al. Dynamic thermal corridor may connect endangered loggerhead sea turtles across the Pacific Ocean. Front Mar Sci. 2021;8(8): 630590.

[46] Howell EA, Dutton PH, Polovina JJ, Bailey H, Parker DM, Balazs GH. Oceanographic influences on the dive behavior of juvenile loggerhead turtles (Caretta caretta) in the North Pacific Ocean. Mar Biol. 2010;157(5):1011–26.

[47] Polovina JJ, Howell E, Parker DM, Balazs GH. Dive-depth distribution of loggerhead (Carretta carretta) and olive ridley (Lepidochelys olivacea) sea turtles in the central North Pacific: Might deep longline sets catch fewer turtles? Fish Bull. 2003;101(1):189–93.

[48] Howell E, Kobayashi D, Parker D, Balazs G, Polovina J. TurtleWatch: a tool to aid in the bycatch reduction of loggerhead turtles Caretta caretta in the Hawaii-based pelagic longline fishery. Endang Species Res. 2008;5:267–78.

[49] Gaspar P, Georges JY, Fossette S, Lenoble A, Ferraroli S, Le Maho Y. Marine animal behaviour: neglecting ocean currents can lead us up the wrong track. Proceed Royal Soc B: Biol Sci. 2006;273(1602):2697–702. 10.1098/rspb.2006.3623 PMC1635505 17015330

[50] Girard C, Sudre J, Benhamou S, Roos D, Luschi P. Homing in green turtles Chelonia mydas: oceanic currents act as a constraint rather than as an information source. Mar Ecol Prog Ser. 2006;20(322):281–9.

[51] Lopez R, Malarde JP, Royer F, Gaspar P. Improving argos doppler location using multiple-model kalman filtering. IEEE Trans Geosci Remote Sens. 2014;52(8):4744–55.

[52] Jonsen ID, Myers RA, James MC. Robust hierarchical state-space models reveal diel variation in travel rates of migrating leatherback turtles: robust hierarchical state-space models. J Anim Ecol. 2006;75(5):1046–57. 16922840 10.1111/j.1365-2656.2006.01129.x

[53] Aijaz S, Brassington GB, Divakaran P, Régnier C, Drévillon M, Maksymczuk J, et al. Verification and intercomparison of global ocean Eulerian near-surface currents. Ocean Model. 2023;186: 102241.

[54] van den Bremer TS, Breivik Ø. Stokes drift. Phil Trans R Soc A. 2018;376(2111):20170104. 29229803 10.1098/rsta.2017.0104 PMC5740299

[55] Beron-Vera FJ, Olascoaga MJ, Miron P. Building a Maxey-Riley framework for surface ocean inertial particle dynamics. Phys Fluids. 2019;31(9): 096602.

[56] Miron P, Olascoaga MJ, Beron‐Vera FJ, Putman NF, Triñanes J, Lumpkin R, et al. (2020) Clustering of Marine‐Debris‐ and *Sargassum* ‐Like Drifters Explained by Inertial Particle Dynamics. Geophysical Research Letters. 47(19): 2020GL089874.

[57] Cunningham HJ, Higgins C, van den Bremer TS. (2022) The Role of the unsteady surface wave‐driven Ekman–stokes flow in the accumulation of floating marine litter. JGR Oceans, 127(6):e2021JC018106.

[58] Higgins C, Vanneste J, van den Bremer TS. 2020 Unsteady Ekman‐Stokes Dynamics: Implications for Surface Wave‐Induced Drift of Floating Marine Litter. Geophysical Research Letters. 47(18):e2020GL089189.

[59] Breivik Ø, Allen AA. An operational search and rescue model for the Norwegian sea and the North sea. J Mar Syst. 2008;69(1–2):99–113.

[60] Maximenko N, Hafner J, Kamachi M, MacFadyen A. Numerical simulations of debris drift from the Great Japan Tsunami of 2011 and their verification with observational reports. Mar Pollut Bull. 2018;132:5–25. 29728262 10.1016/j.marpolbul.2018.03.056

[61] Onink V, Wichmann D, Delandmeter P, van Sebille E. The role of Ekman currents, geostrophy, and stokes drift in the accumulation of floating microplastic. JGR Oceans. 2019;124(3):1474–90. 31218155 10.1029/2018JC014547 PMC6559306

[62] Breivik Ø, Allen AA, Maisondieu C, Roth JC. Wind-induced drift of objects at sea: the leeway field method. Appl Ocean Res. 2011;33(2):100–9.

[63] Lellouche JM, Greiner E, Bourdallé-Badie R, Garric G, Melet A, Drévillon M, et al. The copernicus global 1/12° oceanic and sea ice GLORYS12 reanalysis. Front Earth Sci. 2021;21(9): 698876.

[64] Breivik Ø, Christensen KH. A combined stokes drift profile under swell and wind sea. J Phys Oceanogr. 2020;50(10):2819–33.

[65] Röhrs J, Christensen KH, Hole LR, Broström G, Drivdal M, Sundby S. Observation-based evaluation of surface wave effects on currents and trajectory forecasts. Ocean Dyn. 2012;62(10–12):1519–33.

[66] Poulain PM, Gerin R, Mauri E, Pennel R. Wind effects on drogued and undrogued drifters in the eastern mediterranean. J Atmos Oceanic Tech. 2009;26(6):1144–56.

[67] Duhec AV, Jeanne RF, Maximenko N, Hafner J. Composition and potential origin of marine debris stranded in the Western Indian ocean on remote Alphonse Island. Seychelles Marine Pollution Bulletin. 2015;96(1–2):76–86. 26024564 10.1016/j.marpolbul.2015.05.042

[68] Berline L, Ody A, Jouanno J, Chevalier C, André JM, Thibaut T, et al. Hindcasting the 2017 dispersal of Sargassum algae in the Tropical North Atlantic. Mar Pollut Bull. 2020;158: 111431. 32736205 10.1016/j.marpolbul.2020.111431

[69] Santos BS, Kaplan DM, Friedrichs MAM, Barco SG, Mansfield KL, Manning JP. Consequences of drift and carcass decomposition for estimating sea turtle mortality hotspots. Ecol Ind. 2018;84:319–36.

[70] Law-Chune S, Aouf L, Dalphinet A, Levier B, Drillet Y, Drevillon M. WAVERYS: a CMEMS global wave reanalysis during the altimetry period. Ocean Dyn. 2021;71(3):357–78.

[71] Callies U, Groll N, Horstmann J, Kapitza H, Klein H, Maßmann S, et al. Surface drifters in the German Bight: model validation considering windage and Stokes drift. Ocean Sci. 2017;13(5):799–827.

[72] Christiansen F, Putman N, Farman R, Parker D, Rice M, Polovina J, et al. Spatial variation in directional swimming enables juvenile sea turtles to reach and remain in productive waters. Mar Ecol Prog Ser. 2016;28(557):247–59.

[73] Hays GC, Marsh R. Estimating the age of juvenile loggerhead sea turtles in the North Atlantic. Canadian J Zoology. 1997;75(1):40–6.

[74] Freitas C, Caldeira R, Dellinger T. Surface behavior of pelagic juvenile loggerhead sea turtles in the eastern North Atlantic. J Exp Mar Biol Ecol. 2019;510:73–80.

[75] Laforge A, Gaspar P, Barat A, Boyer JT, Candela T, Bourjea J, et al. Uncovering loggerhead (Caretta caretta) navigation strategy in the open ocean through the consideration of their diving behaviour. J R Soc Interface. 2023;20(209):20230383. 38086403 10.1098/rsif.2023.0383 PMC10715913

[76] Chaloupka M, Parker D, Balazs G. Modelling post-release mortality of loggerhead sea turtles exposed to the Hawaii-based pelagic longline fishery. Mar Ecol Prog Ser. 2004;280:285–93.

[77] Ramirez M, Popovska T, Babcock E. Global synthesis of sea turtle von Bertalanffy growth parameters through Bayesian hierarchical modeling. Mar Ecol Prog Ser. 2021;7(657):191–207.

[78] Lumpkin R, Grodsky SA, Centurioni L, Rio MH, Carton JA, Lee D. Removing spurious low-frequency variability in drifter velocities. J Atmos Oceanic Tech. 2013;30(2):353–60.

[79] Rio MH. Use of altimeter and wind data to detect the anomalous loss of SVP-type drifter’s drogue. J Atmos Oceanic Tech. 2012;29(11):1663–74.

[80] Polovina JJ, Howell E, Kobayashi DR, Seki MP. The transition zone chlorophyll front, a dynamic global feature defining migration and forage habitat for marine resources. Prog Oceanogr. 2001;49(1–4):469–83.

[81] Polovina JJ, Howell EA, Kobayashi DR, Seki MP. The transition zone chlorophyll front updated: advances from a decade of research. Prog Oceanogr. 2017;150:79–85.

[82] Papi F. 1992 Animal homing: general aspects In: Animal Homing. Springer; Berlin p. 1–19

[83] Ishihara T, Kamezaki N, Matsuzawa Y, Iwamoto F, Oshika T, Miyagata Y, et al. Reentery of Juvenile and Subadult Loggerhead Turtles into natal waters of Japan. Current Herpetol. 2011;30(1):63–8.

[84] Virtanen P, Gommers R, Oliphant TE, Haberland M, Reddy T, Cournapeau D, et al. SciPy 1.0: fundamental algorithms for scientific computing in Python. Nat Method. 2020;17(3):261–72. 10.1038/s41592-019-0686-2 PMC7056644 32015543

[85] Bauer S, Nolet BA, Giske J, Chapman JW, Åkesson S, Hedenström A, et al. Cues and decision rules in animal migration. In: Milner-Gulland EJ, Fryxell JM, Sinclair ARE, editors. Animal migration [Internet]. Oxford University Press; 2011 [cited 2024 Apr 18]. p. 68–87. Available from: https://academic.oup.com/book/25920/chapter/193659695

[86] Strandberg R, Klaassen RHG, Hake M, Alerstam T. How hazardous is the Sahara desert crossing for migratory birds? Indications from satellite tracking of raptors. Biol Lett. 2010;6(3):297–300. 19955169 10.1098/rsbl.2009.0785 PMC2880036

[87] Bildstein KL, Bechard MJ, Farmer C, Newcomb L. Narrow sea crossings present major obstacles to migrating Griffon Vultures *Gyps fulvus*. Ibis. 2009;151(2):382–91.

[88] Qiu B. Interannual variability of the Kuroshio extension system and its impact on the wintertime SST field. J Phys Oceanogr. 2000;30(6):1486–502.

[89] Schwartz FJ. Behavioral and tolerance responses to cold water temperatures by three species of sea turtles (Reptilia, Cheloniidae) in North Carolina. Florida Marine Res Publ. 1978;33:16–8.

[90] Seminoff J, Eguchi T, Carretta J, Allen C, Prosperi D, Rangel R, et al. Loggerhead sea turtle abundance at a foraging hotspot in the eastern Pacific Ocean: implications for at-sea conservation. Endang Species Res. 2014;24(3):207–20.

[91] Turner Tomaszewicz CN, Seminoff JA, Avens L, Goshe LR, Peckham SH, Rguez-Baron JM, et al. Age and residency duration of loggerhead turtles at a North Pacific bycatch hotspot using skeletochronology. Biol Cons. 2015;186:134–42. 10.1016/j.biocon.2015.03.015 PMC4384431 25848136

[92] Peckham S, Maldonado Diaz D, Koch V, Mancini A, Gaos A, Tinker M, et al. High mortality of loggerhead turtles due to bycatch, human consumption and strandings at Baja California Sur, Mexico, 2003 to 2007. Endang Species Res. 2008;5:171–83.

[93] Nichols WJ, Resendiz A, Seminoff JA, Resendiz B. Transpacific migration of a loggerhead turtle monitored by satellite telemetry. Bull Mar Sci. 2000;67(3):12.

[94] Matsuzawa Y, Sakamoto W. Effect of incubation temperature on body size of loggerhead hatchlings. Umigame Newslett Japan. 2002;55:17–8.

[95] Bostrom BL, Jones DR. Exercise warms adult leatherback turtles. Comp Biochem Physiol A: Mol Integr Physiol. 2007;147(2):323–31. 17188537 10.1016/j.cbpa.2006.10.032

[96] Durieux G, Liedvogel M. Orientation and navigation in the animal world. In: Morton YTJ, Diggelen F, Spilker JJ, Parkinson BW, Lo S, Gao G, editors. Position, navigation, and timing technologies in the 21st century [Internet]. 1st ed. Wiley; 2020 [cited 2021 Jun 14]. p. 1689–709. Available from: https://onlinelibrary.wiley.com/doi/10.1002/9781119458555.ch54

[97] Mouritsen H. Modelling migration: the clock-and-compass model can explain the distribution of ringing recoveries. Anim Behav. 1998;56(4):899–907. 9790701 10.1006/anbe.1998.0826

[98] Gwinner E. Circadian and circannual programmes in Avian migration. J Exp Biol. 1996;199(1):39–48. 9317295 10.1242/jeb.199.1.39

[99] Mouritsen H. Spatiotemporal orientation strategies of long-distance migrants. In: Berthold P, Gwinner E, Sonnenschein E, editors. Avian migration [Internet]. Berlin, Heidelberg: Springer Berlin Heidelberg; 2003 [cited 2024 Apr 22]. p. 493–513. Available from: http://link.springer.com/10.1007/978-3-662-05957-9_34

[100] Wiltshko W, Wiltshko R. Magnetic compass of European Robins. Science. 1972;176(4030):62–4. 17784420 10.1126/science.176.4030.62

[101] Light P, Salmon M, Lohmann KJ. Geomagnetic orientation of loggerhead sea turtles: evidence for an inclination compass. J Exp Biol. 1993;182(1):1–10.

[102] Åkesson S, Ilieva M, Karagicheva J, Rakhimberdiev E, Tomotani B, Helm B. Timing avian long-distance migration: from internal clock mechanisms to global flights. Phil Trans R Soc B. 2017;372(1734):20160252. 28993496 10.1098/rstb.2016.0252 PMC5647279

[103] Mott CR, Salmon M. Sun compass orientation by juvenile green sea turtles ( *Chelonia mydas* ). Chelonian Conserv Biol. 2011;10(1):73–81.

[104] Able KP. The concepts and terminology of bird navigation. J Avian Biol. 2001;32(2):174–83.

