# Supplementary material for: Swim, baby, swim: the active dispersal scenario of juvenile North Pacific loggerhead turtles revealed by historical satellite tracking data and novel operational oceanography products
Source: Mov Ecol. 2025 Jun 10;13:39. doi: 10.1186/s40462-025-00562-5 (PMC12153208; doi:10.1186/s40462-025-00562-5)
Supplement: Supplementary file 1 — Additional file 1. [file 40462_2025_562_MOESM1_ESM.docx]

**ADDITIONAL FILE 1: SEGMENTATION OF ZONAL DC-TRAJECTORIES**

### Segmentation and classification method

Segmenting animal trajectories as a function of changes in the underlying individual behavior is a classical problem in movement ecology [1], a rather simple one in our case since we look for maximum one change (breakpoint) in one-dimensional trajectories. This breakpoint shall separate an H segment, characterized by a markedly negative mean DC-velocity, from a D segment with a closer-to-zero mean DC-velocity. Formally, each loggerhead trajectory being associated with a time-series of zonal DC-velocities [$u_{DC}\left( t_{i} \right), i=0,N]$ , the segmentation algorithm has to identify the time $t_{k}$ ($0<k<N$) when the mean of $u_{DC}$changes. Detecting, single or multiple, changes in the mean of a time series is a well-studied problem. It can be viewed as equivalent to finding the, single or multiple, step function that best fits this time series in the least-square sense [2]. When searching for a single breakpoint, the problem boils down to finding the index $k$ that minimizes the cost function:

$C\left( k \right)= \sum_{i=0}^{k} {{[u}_{DC}\left( t_{i} \right)-\bar{u_{DC1}}(k)]}^{2}+\sum_{i=k+1}^{N} {{[u}_{DC}\left( t_{i} \right)-\bar{u_{DC2}}(k)]}^{2}$ (A1)

where $\bar{u_{DC1}}(k)$ and $\bar{u_{DC2}}(k)$ are the means of $u_{DC}$between $t_{0}$ and $t_{k}$, and between $t_{k+1}$ and $t_{N}$, respectively. In practice, this problem is easily solved by exhaustive search: for each trajectory, *C* is computed for all values of $k$ and the value that minimizes *C* is selected. With this approach each trajectory is systematically divided in two segments. Each segment is then classified as a H segment if its mean zonal DC-velocity $\bar{u_{DC}}$is smaller than some (negative) threshold velocity $u_{T}$ and as a D segment if $\bar{u_{DC}}>u_{T} .$

### Validation process

The above-described segmentation algorithm always delimits 2 segments in any given trajectory. Each of them is then classified as a D or a H segment. It does not mean that the corresponding segmentation is always appropriate nor that the classification is accurate. Validity of the achieved segmentation and classification must thus be assessed in order to detect unreliable segments (denoted U segments) and eliminate them from any subsequent analysis of the zonal swimming activity.

Segmentation and classification problems mostly arise in short segments and/or inhomogeneous segments. Classification methods typically have problems achieving high accuracy on small data sets. In our case, this happens when a trajectory is short or when a breakpoint is positioned very close to the beginning or the end of a trajectory so that it defines a short segment. In that case, the probability of segment misclassification is large since the uncertainty on the estimated mean DC-velocity increases as the size of the segment decreases. To minimize the risk of misclassification we will thus discard segments that are shorter than 90 days, that is about a season. This suits our purpose well since we only seek to identify long-lasting consistent swimming activities, typically at seasonal or longer time scales. Wild turtle trajectories will be more impacted by this choice than the, generally longer, captive-reared turtle trajectories.

A segmentation is deemed appropriate if it properly separates homogeneous segments from each other. The segment homogeneity criterium is defined in the cost function, the total cost of a complete trajectory being the sum of the costs of each individual segment, as in Eq. (A1). The cost of a homogeneous segment is expected to be low. A high segment cost suggests a segmentation problem, i.e. suboptimal positioning of a breakpoint or inhomogeneous behavior inside the segment possibly requiring further segmentation [3]. In our case, the cost of each segment is simply proportional to the variance of $u_{DC}$ (15). A relatively large variance, or standard deviation, is thus symptomatic of inhomogeneous segments. To identify such segments, segmentation must first be performed.

To do so, we first discard all trajectories shorter than 90 days (n=22) and then segment the other ones (n=210) as described above. We further discard all segments shorter than 90 days (n= 146) and then examine the distribution of the standard deviation and mean of $u_{DC}$ along the 274 remaining segments (Fig. A1).

**
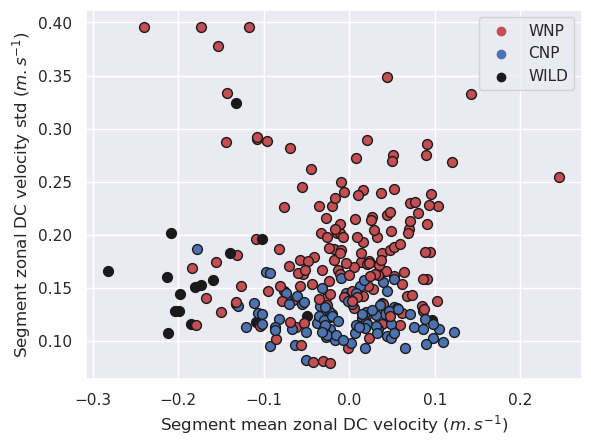
**

**Fig. A1** Scatterplot of the mean and standard deviation of the zonal DC-velocities ($u_{DC})$for the 274 longer-than-90-days trajectory segments. Data for WNP, CNP and wild turtles are plotted in red, blue and black respectively.

This figure clearly shows that high standard deviations are typical of WNP turtles. This is not surprising as estimations errors in DC-velocities are largest near the Japanese coast (Fig. 5b in the main text) where WNP turtles are released. Based on Fig. A1, we choose to discard all segments in which the standard deviation of $u_{DC}$ exceeds 0.25 m/s. With this choice, 22 segments are eliminated, all belonging to turtles released close to the Japanese coast (21 WNP turtles and one wild turtle). Nearly two thirds of the daily positions in these segments are located between the Japanese coast and 160°E. Choosing a smaller standard deviation threshold, would eliminate segments further away from the problematic area near the coast of Japan. At the end of the validation process, we are thus left with 252 valid segments, that is segments longer than 90 days and for which the standard deviation of $u_{DC}$is smaller than 0.25 m/s.

### Estimation of the threshold velocity $\boldsymbol{u}_{\boldsymbol{T}}$using Gaussian Mixture Models

After having selected long and reasonably homogeneous segments, selection of the threshold velocity is the last technical task that remains before these segments can be classified as D or H segments. The histogram of their mean zonal DC-velocities $\bar{u_{dc}}$ (Fig. A2) proves to be markedly asymmetric with a peak close to zero and a large negative tail containing many segments with $\bar{u_{dc}}$< -10 cm/s. The corresponding probability density function $f(\bar{u_{dc}})$ can be fitted by a mixture of Gaussian functions $G_{i}$with means and standard deviations ($\mu_{i}$, $\sigma_{i}$):

$$f\left( \bar{u_{DC}} \right)=\sum_{i=1}^{m} w_{i}G_{i}(\bar{u_{DC}})$$

where m is the number of Gaussian components and $w_{i}$their weights. All weights must be positive and their sum is 1.

**
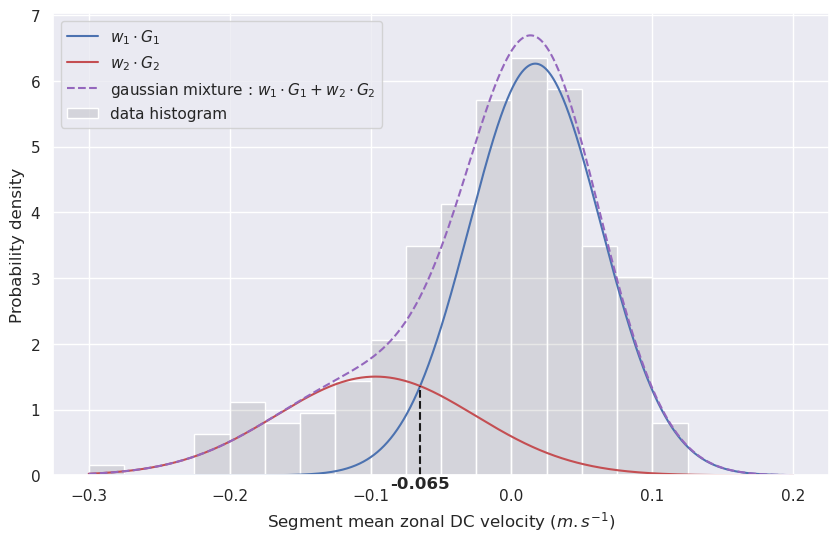
**

**Fig. A2** Normalized histogram of the mean zonal DC-velocities $\bar{u_{DC}}$in the 252 validated segments of turtles’ DC-trajectories together with the components of the Gaussian Mixture Model that best approximates this histogram.

Using the GaussianMixture object of the Scikit-Learn Python library [3] maximum likelihood estimates of the parameters ($w_{i}, \mu_{i}$, $\sigma_{i}$) are obtained via the EM algorithm [4]. This is done for increasing values of m, starting with m=1. The corresponding Bayesian Information Criterium (BIC) is computed. Interestingly, the value of m that minimizes the BIC is m=2, indicating that $f(\bar{u_{dc}})$ is best fitted by a mixture of two Gaussian functions which parameters are given in Table A1.

*Table A1 Parameters of the selected Gaussian Mixture Model*

| **Component** | **Weight** | **Mean (m/s)** | | **Std (m/s)** |
| --- | --- | --- | --- | --- |
| $G_{1}$ | 0.734 | 0.017 | 0.216 | |
| $G_{2}$ | 0.266 | -0.096 | 0.266 | |

This result supports the idea that the previously identified segments belong to two populations which DC-velocities follow different Gaussian distributions. The first population, characterized by the $G_{1}$Gaussian distribution, has a small mean DC-velocity (1.7 cm/s) and corresponds to the population of D segments. The second population ($G_{2}$) with a large negative mean DC-velocity (-9.7 cm/s), is the population of H segments. Following the usual GMM classification approach, we choose $u_{T}$ = -6.5 cm, the value of $\bar{u_{DC}}$ for which the probability of belonging to one population becomes larger/smaller than the probability of belonging to the other. Final results of the above-described segmentation/classification procedure using $u_{T}$= -6.5 cm/s are presented in the main text.

**REFERENCES**

1. Edelhoff H, Signer J, Balkenhol N. Path segmentation for beginners: an overview of current methods for detecting changes in animal movement patterns. Mov Ecol. déc 2016;4(1):21.

2. Yao YC, Au ST. Least-Squares Estimation of a Step Function. Sankhyā Indian J Stat Ser 1961-2002. 1989;51(3):370‑81.

3. Pedregosa F, Varoquaux G, Gramfort A, Michel V, Thirion B, Grisel O, et al. Scikit-learn: Machine Learning in Python. J Mach Learn Res. 2011;12(85):2825‑30.

4. Dempster AP, Laird NM, work(s): DBRR. Maximum Likelihood from Incomplete Data via the EM Algorithm. J R Stat Soc Ser B Methodol. 1977;39(1):1‑38.
